# *Plasmodium* Impairs Antibacterial Innate Immunity to Systemic Infections in Part Through Hemozoin-Bound Bioactive Molecules

**DOI:** 10.3389/fcimb.2020.00328

**Published:** 2020-06-30

**Authors:** Christopher L. Harding, Nicolas F. Villarino, Elena Valente, Evelin Schwarzer, Nathan W. Schmidt

**Affiliations:** ^1^Department of Microbiology and Immunology, University of Louisville, Louisville, KY, United States; ^2^Department of Veterinary Clinical Sciences, Washington State University, Pullman, WA, United States; ^3^Department of Oncology, University of Torino, Turin, Italy; ^4^Ryan White Center for Pediatric Infectious Diseases and Global Health, Department of Pediatrics, Indiana University School of Medicine, Indianapolis, IN, United States

**Keywords:** *Plasmodium*, malaria, immunosuppression, co-infection, bacteria, innate immunity, pneumococcus

## Abstract

One complication of malaria is increased susceptibility to invasive bacterial infections. *Plasmodium* infections impair host immunity to non-Typhoid *Salmonella* (NTS) through heme-oxygenase I (HO-I)-induced release of immature granulocytes and myeloid cell-derived IL-10. Yet, it is not known if these mechanisms are specific to NTS. We show here, that *Plasmodium yoelii* 17XNL (Py) infected mice had impaired clearance of systemic *Listeria monocytogenes* (Lm) during both acute parasitemia and up to 2 months after clearance of Py infected red blood cells that was independent of HO-I and IL-10. Py-infected mice were also susceptible to *Streptococcus pneumoniae* (Sp) bacteremia, a common malaria-bacteria co-infection, with higher blood and spleen bacterial burdens and decreased survival compared to naïve mice. Mechanistically, impaired immunity to Sp was independent of HO-I, but was dependent on Py-induced IL-10. Splenic phagocytes from Py infected mice exhibit an impaired ability to restrict growth of intracellular Lm, and neutrophils from Py-infected mice produce less reactive oxygen species (ROS) in response to Lm or Sp. Analysis also identified a defect in a serum component in Py-infected mice that contributes to reduced production of ROS in response to Sp. Finally, treating naïve mice with *Plasmodium*-derived hemozoin containing naturally bound bioactive molecules, excluding DNA, impaired clearance of Lm. Collectively, we have demonstrated that *Plasmodium* infection impairs host immunity to diverse bacteria, including *S. pneumoniae*, through multiple effects on innate immunity, and that a parasite-specific factor (Hz+bound bioactive molecules) directly contributes to *Plasmodium*-induced suppression of antibacterial innate immunity.

## Introduction

Despite efforts to decrease the global health burden of malaria, infections with *Plasmodium* species continue to cause over 200 million episodes of malaria each year (Organization WH, 2018). These infections resulted in 435,000 deaths in 2017 the majority of which occur in sub-Saharan Africa in children under the age of 5 years (Organization WH, 2018). In areas with endemic malaria, an association between *Plasmodium* infection and invasive bacterial infections has been reported, and malaria was identified as a major risk factor for developing bacteremia that correlated with a worse clinical outcome and higher morbidity (Berkley et al., 2005; Reddy et al., 2010; Colombatti et al., 2011; Scott et al., 2011; Were et al., 2011; Auma et al., 2013; Gomez-Perez et al., 2014; Morton et al., 2018). These reports implicate both gastrointestinal and pulmonary bacterial pathogens such as non-typhoid *Salmonella* (NTS) and *Streptococcus pneumoniae* (Sp), respectively, as the most common organisms isolated from blood of *Plasmodium* and bacteria co-infected individuals (Berkley et al., 2005; Reddy et al., 2010; Scott et al., 2011; Decuypere et al., 2016; Mourembou et al., 2016).

Recent studies have characterized some of the underlying mechanisms by which *Plasmodium* infections increase susceptibility to invasive NTS infection (Cunnington et al., 2011, 2012; Lokken et al., 2014, 2018; Mooney et al., 2014, 2018). First, *Plasmodium*-induced hemolysis results in the activation of heme oxygense-1 (HO-1), which mobilizes functionally immature granulocytes from the bone marrow. These immature granulocytes exhibited impaired production of reactive oxygen species in response to PMA stimulation, and decreased killing of NTS (Cunnington et al., 2011). Similar impairments to NTS immunity were observed when mice were treated with phenylhydrazine to induce hemolysis or hemin to mimic hemolysis, leading to the conclusion that impaired bacterial immunity during *Plasmodium* infection is attributed to hemolysis with no parasite-specific factor contributing to immune suppression (Cunnington et al., 2012). Second, *Plasmodium*-induced IL-10 from myeloid cells suppresses the ability of myeloid cells to control NTS, resulting in increased systemic colonization in co-infected mice (Lokken et al., 2014). Moreover, IL-10 signaling can directly induce HO-1 to suppress anti-bacterial immunity, creating an environment that favors intracellular growth and dissemination of NTS (Lee and Chau, 2002). Importantly, it is not known whether these mechanisms are unique to NTS co-infections versus mechanisms that are generally applied to other systemic bacterial infections. Furthermore, no parasite-specific factor has been identified to contribute toward *Plasmodium*-induced impaired immunity to bacterial co-infections. These represent important knowledge gaps that impede treatment of bacteria co-infections during malaria.

Digestion of hemoglobin results in the release of heme, which is toxic to the parasite. Heme is detoxified within the parasite's digestive vacuole via non-enzymatic polymerization into hemozoin (Hz), which is an insoluble crystal structure. Following erythrocyte rupture, Hz is released into circulation and engulfed by phagocytic cells resulting in deposition in tissues and organs such as spleen, liver, brain, lungs, and bone marrow. Hz deposition following *Plasmodium berghei* infection was evident in the spleen, liver, bone marrow, and peripheral blood 196 days after infection (Frita et al., 2012). Hz has also been reported in lung and brain tissue of *P. berghei* and *Plasmodium chabaudi* infected mice (Deroost et al., 2012). The immune modulatory potential of Hz has been examined in several studies with a wide variety of Hz preparations and cell types being used. While synthetically generated Hz has been described in some cases as “immunologically inert,” others have found Hz formed naturally during erythrocyte infection to alter and impair many immune cell functions (Parroche et al., 2007). For example, uptake of parasitized red blood cells (RBCs) or purified Hz by human monocyte-derived macrophages causes impaired phagocytosis of additional RBCs or production of a PMA-induced oxidative burst (Schwarzer et al., 1992), which are impaired via Hz-dependent inactivation of protein kinase C and NADPH-oxidase (Schwarzer et al., 1993; Schwarzer and Arese, 1996). Internalization of Hz has also been found to induce the production of pro-inflammatory cytokines such as IL-1β and TNF and also increased chemokine production and enhanced expression of their receptors (CCL3-5, MCP-1) in mouse macrophages and human peripheral blood monocytes (Sherry et al., 1995; Giribaldi et al., 2010). Antigen presentation functions and overall functional maturation of dendritic cells are also impacted, as decreased surface expression of CD54, CD11c, and MHC-class II in human PBMCs following stimulation with IFN-γ has also been observed during co-incubation with Hz (Schwarzer et al., 1998). Few studies, however, have looked directly at the interactions between innate immune cells and Hz in the context of *in vivo* bacterial infection or the effect of Hz on neutrophil function. Moreover, previous work has shown impaired antimicrobial functions of innate immune cells from African children with uncomplicated malaria that lasted up to 8 weeks after antimalarial treatment (Cunnington et al., 2012). Of note, there is unlikely to be parasite-induced hemolysis during convalescence, which is the current hypothesis by which *Plasmodium* suppresses anti-bacterial innate immunity. Collectively, this leads to the hypothesis that Hz contributes to *Plasmodium*-induced suppression of innate immunity to bacterial infections.

We demonstrate here, a long-lasting defect in the clearance of *Listeria monocytogenes* (Lm) from spleens of mice previously infected with *Plasmodium yoelii* 17XNL (Py). This effect was independent of IL-10 and HO-1. Py also induced long-lasting impaired immunity to Sp, which resulted in decreased survival compared to control mice. Py impaired control of Sp was dependent on IL-10, but independent of HO-1. In addition to the *in vivo* infections, we show that splenic neutrophils from Py-infected mice have impaired ROS production in response to both Lm and Sp, with a defective serum factor further decreasing this response to Sp. Finally, we demonstrate that “native” Hz isolated from Py convalescent mice (PyHz), defined as Hz containing naturally associated bioactive molecules, such as host and parasite-derived proteins and lipids but not DNA, significantly impaired the ability of splenic phagocytes to control growth of intracellular Lm. Both PyHz and “native” Hz isolated from *P. falciparum*-infected red blood cell cultures (PfHz) were sufficient to impair clearance of Lm *in vivo*. Thus, we identified a parasite-specific factor that directly contributes to impaired immunity to a systemic bacterial infection.

## Materials and Methods

### Mice

Female C57BL/6N mice 6–12 weeks old were obtained from Charles River Laboratories (Wilmington, MA) and housed at the University of Louisville under appropriate biosafety level conditions. All mice were housed in a specific pathogen-free facility and acclimatized for a minimum of 7 days before starting experiments. Animals were fed the NIH-31 diet (Modified Open Formula Mouse/Rat Irradiated Diet; Harlan 7,913; Envigo, Indianapolis, IN) and provided autoclaved, non-acidified reverse-osmosis water ad libitum. The mice were kept on a 12-h light/dark cycle from 6 AM to 6PM and 6PM to 6AM, respectively. All animal handling and experimentation were reviewed and approved by the University of Louisville Institutional Animal Care and Use Committee based on the recommendations of the Guide for the Care and Use of Laboratory Animals of the National Institutes of Health.

### Infections

#### Plasmodium yoelii

Mice were infected i.v. with 5 × 10^6^ parasitized red blood cells (pRBC) *Plasmodium yoelii* 17XNL (Py). Parasitemia (% of infected red blood cells) was determined by analyzing samples of peripheral blood obtained from day 5 post Py infection and alternating days afterward until mice cleared detectable infected RBCs. Blood samples were fixed in 0.02% glutaraldehyde and stained with antibodies specific to CD45-APC (clone 104) and Ly-76 (clone Ter119) from Biolegend (San Diego, CA) and dyes dihydroethidium, and Hoechst 33342 (Sigma-Aldrich, St. Louis, MO). Parasitemia was analyzed with flow cytometry using a BD Fortessa, where pRBCs were identified as the population of CD45^−^, Ter119^+^, dihydroethidium^+^, Hoechst^+^ cells. All flow cytometry data was analyzed with FlowJo software.

#### Listeria monocytogenes

*actA*-deficient *Listeria monocytogenes* strain 10403S was grown from freezer stock aliquot in tryptic soy broth and then washed with PBS and resuspended in sterile saline. Mice were infected i.v. with 5 × 10^6^ CFU on indicated days post Py infection or as an initial infection in age-matched mice.

#### Streptococcus pneumoniae

The following reagent was obtained through the NIH Biodefense and Emerging Infections Research Resources Repository, NIAID, NIH:

*Streptococcus pneumoniae* (Sp) ATCC strain 6303 a type 3 capsule isolate. Cultures were started from freezer stocks and grown in brain heart infusion broth containing 50 mM HEPES buffer and incubated statically at 37°C with 5% CO_2_. Cultures were washed in PBS and resuspended in sterile saline. Mice were infected with 1 × 10^6^ CFU of Sp intranasally to naïve mice or Py infected mice on day seven post Py infection in a volume of 30 μl. Inoculum dose was confirmed by plating serial dilutions on blood agar and counting colonies after 12–18 h of growth at 37°C with 5% CO_2_.

### Bacterial Burden Quantification Analysis

Spleens and livers from Lm infected mice were collected in 4 ml 0.2% IGEPAL (Sigma Aldrich, St. Louis, MO) at the indicated timepoints. All organs were homogenized with a tissue master homogenizer (Omni International, Kennesaw GA) and plated in serial dilutions on tryptic soy agar plates with 50 μg/mL streptomycin. Lungs and spleens from Sp infected mice were collected in sterile 1X PBS. Organs were homogenized and plated in serial dilutions on tryptic soy agar plates supplemented with 10% citrated sheep blood (Hardy Diagnostics, Springboro, Ohio). Peripheral blood was collected via retro-orbital bleed and plated directly on tryptic soy agar plates with 10% sheep blood agar.

### *In vivo* Inhibition of IL-10 and HO-1

Py-infected and naïve mice were injected i.p. with 300, 200, and 100 μg of rat anti-IL-10 IgG1 kapa monoclonal antibody (clone JES5-2A5) or rat anti-horseradish peroxidase IgG1 isotype control (In*Vivo*MAb, West Lebanon, NH) in 0.2 mL sterile saline on days Py+6, 7, and 8 for Lm co-infection (day Lm −1, 0, and +1), days Py+7, 8, and 9 for Sp co-infection (day Sp −1, 0, and +1). Similarly, Py-infected and naïve mice were treated i.p. with 40 μmol/kg Tin Protoporphyrin IX (chloride) (Sn PP) (Cayman Chemical, Ann Arbor, MI) in 0.2 mL 1X PBS on days Py+5, 6, 7, and 8 for Lm co-infection (day Lm −2, −1, 0, and +1), days Py+6, 7, 8, and 9 for Sp co-infection (day Sp −2, −1, 0, and +1).

### Splenic Myeloid Cell Analysis

Spleens were removed on the indicated days post Py infection or from age-matched naïve mice and placed in HyClone RPMI 1640 (Thermo Fisher Scientific, Waltham, MA) supplemented with 10% fetal bovine serum (Atlanta Biologicals, Lawrenceville, GA), 1.19 mg/ml HEPES (Corning, New York, NY), 0.2 mg/ml L-glutamine (Research Products International, Mt. Prospect, IL), 0.05M 2-mercaptoethanol (Thermo Fisher Scientific), 0.05 mg/mL penicillin/streptomycin (Invitrogen, Grand Island, NY), and 0.05 mg/mL gentamicin (Corning). Single cell suspensions were generated by manually disrupting spleens with the plunger of a 5 mL syringe over fine a wire mesh and then treated with ammonium chloride potassium solution (0.15 mM NH_4_Cl, 1.0 mM KHCO_3_, 0.1 mM Na_2_ EDTA, 1 N HCl) to lyse red blood cells and then washed in RPMI then resuspended in FACS buffer (1XPBS, 1%FBS, 0.02% sodium azide) containing Fc block (anti-CD16/32; clone 2.4G2). Splenocytes were then stained with cell surface marker-specific antibodies. Anti-CD3ϵ FITC (clone 145-2C11), anti-CD19 FITC (clone 6D5), anti-CD11b PE/Cy7 (clone M1/70), anti-CD11c BV650 (clone N418), anti-Ly-6G BV421 (clone 1A8), anti-Ly-6C PerCP/Cy5.5 (clone AL-21), anti-F4/80 PE Dazzle 594 (clone BM8) were from Biolegend (San Diego, CA). Anti-CD209b APC (clone eBio 22D1), anti-CD169 (clone SER-4) were from eBiosciences (San Diego, CA). Anti-SIGLEC-F APC R700 (clone E50-2240) was from BD Pharmingen (San Diego, CA). Cells were incubated with antibodies at 4°C for 15 min. Cells were then fixed in 4% paraformaldehyde fixation buffer (Biolegend) and then washed three times in FACS buffer before being analyzed on a BD LSRFortessa.

### Spleen Phagocytic Cell Isolation and Intracellular Bacterial Survival Assay

Single cell suspensions from 3 naïve or Py infected mice were generated as described above and pooled together. Splenic phagocytes were enriched by negative depletion of CD3^+^ and CD19^+^ cells using anti-CD3ϵ and anti-CD19 microbeads (Miltenyi Biotec, Bergisch Gladbach, Germany), according to manufacturer recommendations. CD3^−^ and CD19^−^ flow through cells were stained with anti-CD11b PE/Cy7 (clone M1/70) and CD11c APC (clone N418) from Biolegend (San Diego, CA). CD11b^hi^/CD11c^−^ cells were sort purified with a BD FACSAria III. Sorted cells were plated and incubated at 37°C with 5% CO_2_ for 1 h. For some experiments, cells from naïve mice were incubated for 30 min with 30 μg Py hemozoin (PyHz). Sorted cells were infected with Lm that had been opsonized by incubating in RPMI with 10% homologous mouse serum (naïve cells + Lm opsonized with naïve serum; Py cells + Lm opsonized with Py serum) rotating for 30 min at 37°C. Bacteria was washed once in RPMI and cells were infected with a multiplicity of infection (MOI) = 100. One hour after the infection, media was removed from cells and replaced with RPMI containing 5 μg/mL gentamicin to kill extracellular Lm. Intracellular Lm burden was determined after 1 and 4 h of incubation in RPMI with gentamicin by removing the media containing gentamicin and resuspending the cells in sterile deionized H_2_O (dH_2_O) to lyse cells and release surviving Lm. Fold change in intracellular Lm was determined by dividing cfu at 4 h post infection by cfu/well at 1 h after the addition of gentamicin.

### Microscopy

Cells were from either peripheral blood, obtained through tail snip, or spleen or liver single cells isolated and passed through a 100-micron pore size nylon mesh. In some experiments, CD11b^hi^/CD11c^−^ cells were sort purified from T cell and B cell depleted spleen samples. Blood samples were smeared on a glass slide and allowed to dry, spleen and liver cells were placed onto a glass slide in a drop of RPMI containing 15% mouse serum and incubated at 37°C with 5% CO_2_ for 30 min to allow cells to adhere. For all samples, cells were fixed in methanol for 20 min and stained with Giemsa dye (Azer Scientific, Morgantown, PA) for 20 min and rinsed with ddH_2_O. In some cases, cells were incubated with Hz as described in above and Hz is visible as brown pigment, indicated with arrows. Images were obtained with a Nikon eclipse Ci light microscope at 100X magnification or a Zeiss Axio Imager. A1 microscope with an AxioCam MRm digital camera (Zeiss) at 63 or 96X in oil emersion. Scale bars were estimated based on average diameter of murine erythrocytes (Nikon) or with Axiovision SE64 imaging software (Carl Zeiss Microscopy, LLC, White Plains, NY).

### Isolation of *P. yoelii*-Derived Hemozoin (PyHz)

Livers and spleens were obtained from mice previously infected with Py after resolution of acute hyperparasitemia, between 30- and 65-days post-infection. Organs were collected and homogenized with a tissue homogenizer in de-ionized H_2_O (dH_2_O) and kept on ice for 1 h. Homogenates were centrifuged at 3,100 × g for 10 min. Pellets were washed twice in dH_2_O and passed through 100-micron nylon mesh (ELKO Filtering Co.). Flow-through was collected, washed and resuspended in sterile PBS before being added to the top of a discontinuous Percoll density gradient containing 40% (1.07 g/mL) and 80%(1.12 g/mL) fractions. Gradients were centrifuged at 5,000 × g for 20 min with slow acceleration/deceleration at 4°C. Hemozoin from Py infected mouse organs (PyHz) was collected from the interphase between the 40 and 80% fractions, as well as from the dark pellet beneath the 80% fraction. Samples were washed twice in dH_2_O and finally resuspended in sterile PBS. Purification of “clean” PyHz was performed for some experiments with a method adapted from Coban et al. (2002), where organ homogenate was lysed in 0.2% IGPAL and washed 3–4 times in 2% sodium dodecyl sulfate (SDS) and then incubated overnight in 10 mM Tris-HCl (pH 8.0), 0.5% SDS, 1 mM CaCl_2_, and 2 mg/mL proteinase K at 37°C. Next, samples were washed again in 2% SDS and then incubated at 37°C for 3 h in 6M urea, washed in 1X PBS and resuspended in saline for experimental use. For quantification of Hz in mouse tissues, organs were homogenized and incubated in 1% Proteinase K (VWR, Solon, OH) overnight at 37°C. Hz was quantified using a protocol adapted from Deroost et al. (2012). Briefly, Hz was centrifuged at 3,100 × g and pellets were washed three times in 100 mM NaHCO_3_, pH 9.0 with 2% SDS. Hz, and a dilution series of synthetic hemozoin (InVivoGen, San Diego, CA) of known concentration, were resuspended in 100 mM NaOH, 2% SDS, 3 mM EDTA and then sonicated for 5 min before incubating for 30 min at room temp to dissolve hemozoin into soluble heme. Quantification was determined by reading absorbance at OD_400_ and comparing values of samples prepared from Py-infected mouse organs to the synthetic Hz standards. For some experiments, PyHz was incubated at 37°C in 100 U/mL DNase with 1 mM CaCl_2_ and 2 mM MgCl_2_ for 2 h. These samples were then washed twice in 2 mM mannitol-containing phosphate buffer and resuspended in sterile saline. Absence of DNA in PyHz sample was verified by staining with GelRed Nucleic Acid stain and running samples on 1% agarose gel.

### *Plasmodium falciparum* Culture and Preparation of Hemozoin

Natural *P. falciparum* hemozoin (PfHZ) was prepared from *in vitro* human erythrocytes cultures infected with *P. falciparum* (Palo Alto strain, mycoplasma-free) treated with DNase (Sigma, St. Louis, MO). Contamination with endotoxin (LPS) was excluded by E-Toxate assay (Sigma, St. Louis, MO), as previously described (Barrera et al., 2011). Briefly, synchronized parasite cultures were supplemented with DNase (5 U/ml) 12–15 h previous to schizogony. PfHZ which was naturally expelled during schizogony, was harvested at the 25/40% interface of a discontinuous percoll gradient, incubated with 40 U/ml DNase, 2 mM MgCl_2_ and 1 mM CaCl_2_ in PBS at 37°C for 1 h and subsequently washed several times in a 10 mM phosphate buffer (pH 8.0) containing 2 mM mannitol. HZ was quantified by its heme content using a luminol-enhanced luminescence assay as previously described (Schwarzer et al., 1994).

### Reactive Oxygen Species Assay

Spleens and blood (for opsonizing bacteria) were collected from naïve control and Py-infected mice (days 10–16 post infection). Spleens were processed to generate single cell suspensions. Cells were plated at 2 × 10^6^ cells/well resuspended in RPMI with 10% fetal calf serum incubated at 37°C for 30 min with 5% CO_2_. Cells were then incubated with the following antibodies; anti-CD3ϵ APC (clone 145-2C11), anti-CD19 APC (clone 6D5), anti-CD11b PE/Cy7 (clone M1/70), anti-Ly-6G BV421 (clone 1A8), and 2′,7′-dichloro-hydrofluorescein diacetate (DCF; Abcam Cambridge, MA) for 20 min. Cells were then left untreated or treated for 20 min at 37°C with 5% CO_2_ with 20 μM phorbol 12-myristat 13-acetate (PMA), unopsonized Lm or Sp, naive serum opsonized Lm or Sp, or Py serum opsonized Lm or Sp at a MOI of 10. Sp was heat treated (Ht Sp) at 65°C for 1 h. Lm and Ht Sp were opsonized in 10% serum, from naïve or Py-infected mice, in RPMI while incubating at 37°C and rotating. After treatment, cells were centrifuged at 300 x g for 5 min then incubated at 37°C for 20 min followed immediately by analysis on a BD LSRFortessa.

### Statistical Analysis

Data were analyzed as indicated in each figure legend using GraphPad Prism v7.

## Results

### *P. yoelii* Infection Induces Long-Term Susceptibility to Bacterial Infections

**Figure 1:**
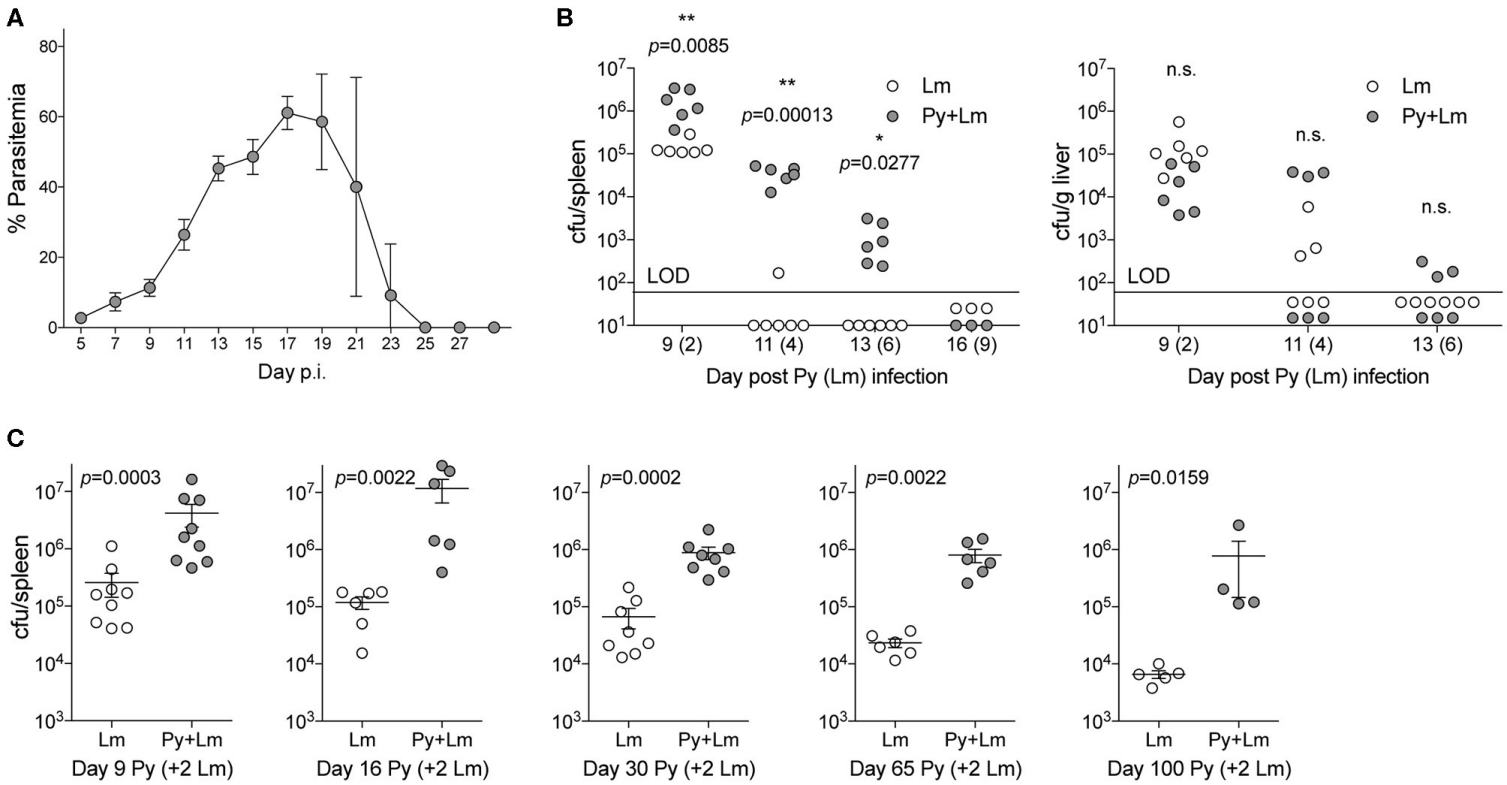
*P. yoelii* infection induces long-term suppression of anti-bacterial innate immunity during bacteremia. **(A)** C57BL/6N mice were infected with 10^5^ Py pRBCs. Percent parasitemia (percent of RBCs infected with Py) was evaluated during the course of infection. Data (mean ± S.D.) are from nine mice and representative of three independent experiments. **(B)** C57BL/6N mice were infected with 10^5^ Py pRBCs, 7 days later both control and Py-infected mice were infected with 5 × 10^6^ CFU Lm. Lm burden was determined in the spleen and liver on days 2, 3, 6, and 9 post-Lm infection. Data are cumulative results from two-independent experiments with three or six mice per group per time point. Each symbol represents an individual mouse. Data were analyzed by two-tailed Fisher's exact test. LOD, limit of detection. [cfu/spleen day 11 (White et al., 2015) data was originally published in *The Journal of Immunology*. White C.E., Villarino N.F., Sloan S.S., Ganusov V.V., and Schmidt N.W. 2015. *Plasmodium* Suppresses Expansion of T Cell Responses to Heterologous Infections. *J. Immunol*. 194: 697-708. Copyright © 2015 The American Association of Immunologists, Inc.] **(C)** C57BL/6N mice were infected with 10^5^ Py pRBCs. On days 7, 14, 28, 63, and 98 post-Py infection both Py-infected and control mice were infected with 5 × 10^6^ CFU Lm. Bacterial burdens were determined 2 days post Lm infection. Data (mean ± S.E.) are cumulative results from 2- to 3-independent experiments with 4–9 mice per group. Data were analyzed with Mann-Whitney test, n.s., not significant.

To examine how *Plasmodium* may suppress innate immunity during systemic bacterial infection, C57BL/6N mice were first infected with Py that resulted in increasing parasitemia through about 3-weeks post infection that was followed by resolution of detectable infected RBCs during the ensuing week (Figure 1A). Control and Py-infected mice were infected intravenously with the model bacterium Lm 7-days post Py infection followed by analysis of bacterial burden day 2, 4, 6, and 9 post-Lm infection. Clearance of Lm was delayed, in particular in the spleen, in Py-infected mice compared to control mice (Figure 1B). To further examine increased susceptibility to Lm over the duration of Py-infection, mice were infected with Lm on day 7, 14, and 28 post-Py infection and spleens were removed 2-days later to quantify Lm burden. Throughout the course of Py infection, mice had elevated bacterial burdens (Figure 1C). Remarkably, increased bacterial burden was also observed at both 1-month (day 63) and 2-months (day 98) post-clearance of Py infected RBCs (Figure 1C). Collectively, these data demonstrate that Py impairs control of Lm infection, which lasts well beyond the clearance of infected RBCs from peripheral circulation.

**Figure 2:**
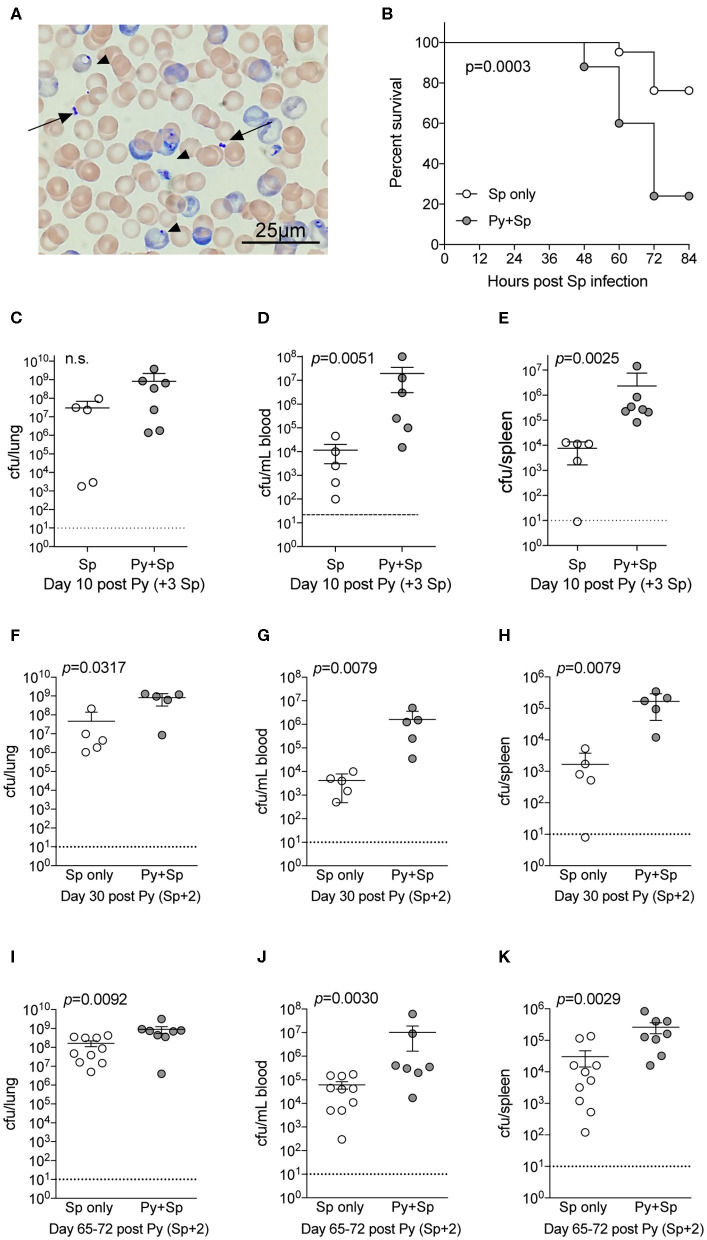
*P. yoelii* infection increases severity of Pneumococcal infection. C57BL/6N mice were infected (i.v.) with 10^5^ *P. yoelii* infected RBCs, 7-days later Py-infected and naïve mice were infected (i.n.) with 10^6^ CFU *Streptococcus pneumoniae* (Sp). **(A)** Giemsa stained blood smear from co-infected mouse. Arrows indicate Sp diplococci, arrow heads indicate *P. yoelii* infected RBCs. **(B)** Survival of mice following Sp infection. Data are pooled results from four independent experiments with a total of 21 Sp only mice and 25 Py+Sp co-infected mice. Survival data was analyzed by log-rank (Mantel-Cox) test. **(C–E)** Mice were sacrificed at either day 3 post Sp infection (10 days post Py infection) or when mice became moribund. Sp burden was quantified in lungs **(C)**, blood **(D)**, and spleen **(E)**. **(C–E)** Data (mean ± S.D.) are from a single experiment that is representative of 3 independent experiments with 5–7 mice per group. **(F–K)** C57BL/6N mice were infected with 10^5^ *P. yoelii* infected RBCs, then infected with 10^6^ CFU Sp either 30 **(F–H)** or 65–72 **(I–K)** days later. Sp bacterial burdens in blood and organs were obtained from mice sacrificed 2 days after Sp infection. **(F–H)** Data (mean ± S.D.) representative of 2 independent experiments with 5–7 mice per group. **(I–K)** Data (meat ± S.E.) cumulative results from two independent experiments with 7–10 mice per group. **(C–K)** Dotted lines indicate limit of detection. Each symbol represents a single mouse. Statistical significance was determined with Mann-Whitney test.

*Streptococcus pneumoniae* (Sp) is one of the most common bacteria observed in individuals co-infected with malaria and an invasive bacterial infection (Reddy et al., 2010; Scott et al., 2011). To formally test the ability of *Plasmodium* to impair control of Sp, mice were infected with Py followed by intranasal Sp infection 7-days later. Following infection, diplococci were clearly evident in the blood of co-infected mice (Figure 2A), which showed significantly lower survival through 84-h post Sp infection compared to control Sp-only infected mice (*p* = 0.0003; Figure 2B). Consistent with the decreased survival of Py-infected mice following Sp infection, bacterial burdens trended higher in the lungs (Figure 2C) and were significantly higher in both the blood (Figure 2D) and spleen (Figure 2E). Mice infected with Sp on day 30 post-Py infection (Figures 2F–H) or day 65–72-post Py infection (Figures 2I–K) had increased bacterial burdens in lungs, peripheral blood, and spleens compared to age matched mice infected with only Sp. These data demonstrate that Py infection also impairs control of Sp bacteremia, and that the *Plasmodium*-driven susceptibility to Sp is prevalent beyond clearance of infected RBCs from peripheral circulation. These observations led to the hypothesis that Py was impairing one or more aspects of antibacterial innate immunity that results in impaired control of these systemic infections.

### Impaired Anti-bacterial Immunity Does Not Correlate With Changes in Splenic Myeloid Populations

**Figure 3:**
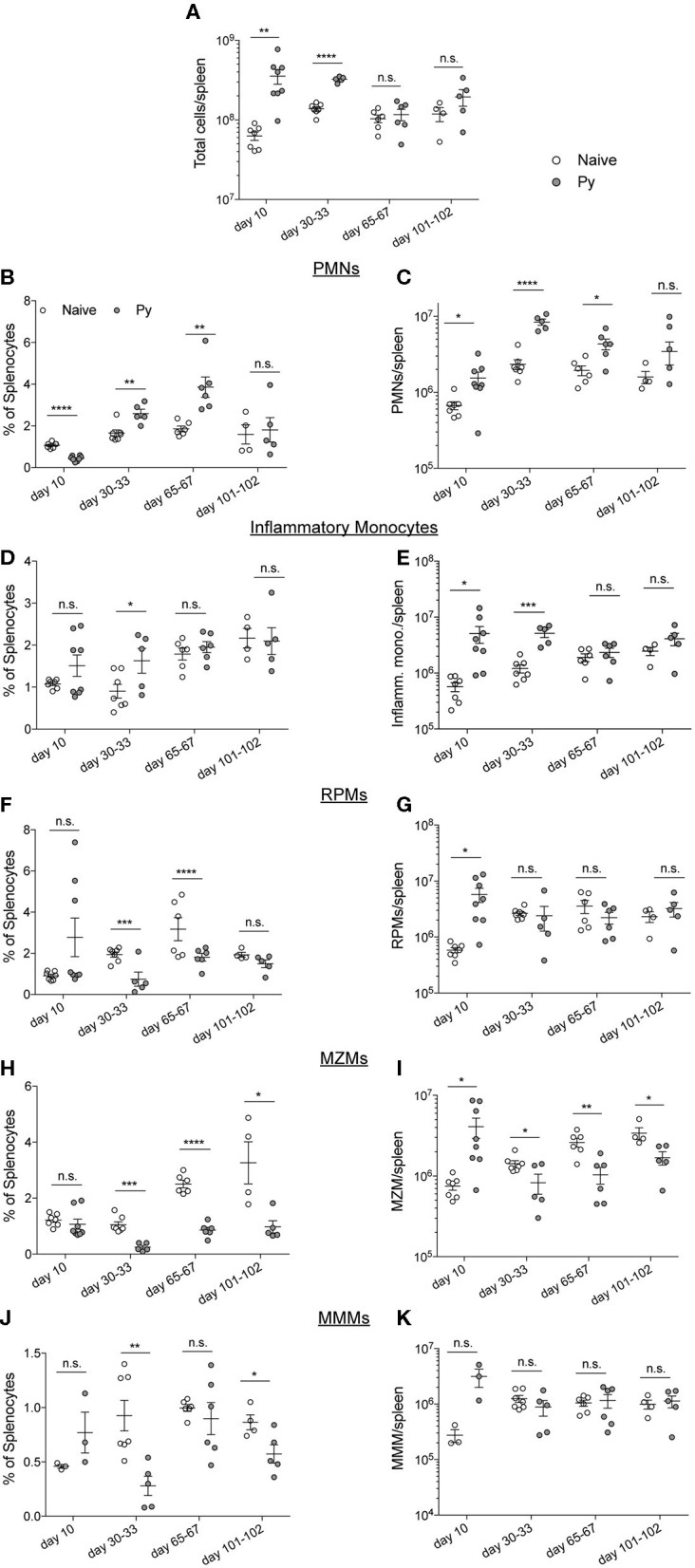
*P. yoelii*-induced temporal changes in splenic myeloid cell populations do not correlate with impaired immunity during bacteremia. C57BL/6N mice were infected with 10^5^ Py pRBCs. Spleens were collected from control or Py-infected mice on the indicated days post infection. **(A)** Total numbers of cells per spleen on the indicated days. Myeloid cell populations were defined as follows: PMNs (CD3^−^, CD19^−^, F4/80^−^, SIGLEC^−^, CD11b^+^, Ly-6G^+^) **(B,C)**, inflammatory monocytes (CD3^−^, CD19^−^, F4/80^−^, SIGLEC^−^, Ly-6G^−^, CD11b^+^, CD11c^−^, Ly-6C^hi^) **(D,E)**, RPMs (CD3^−^, CD19^−^, F4/80^hi^) **(F,G)**, MZMs (CD3^−^, CD19^−^, F4/80^−^, SIGLEC^−^, Ly-6G^−^, CD11b^+^, CD11c^−^, Ly-6C^lo^, CD209b^−^, CD169^+^) **(H,I)**, MMMs (CD3^−^, CD19^−^, F4/80^−^, SIGLEC^−^, Ly-6G^−^, CD11b^+^, CD11c^−^, Ly-6C^lo^, CD169^−^, CD209b^+^) **(J,K)**. **(B,D,F,J)** Percent of splenocytes that are the indicated myeloid cell population. **(C,E,G,I,K)** Total number of indicated myeloid cell population per spleen. Data (mean ± S.E.) are combined results from 1* to 3-independent experiments per timepoint with 3–5 mice per group analyzed with *t*-tests. *Analysis of MMMs on day 10 post Py infection is a single experiment. *p < 0.05, **p < 0.01, ***p < 0.001, and ****p < 0.0001.

*Plasmodium chabaudi* infections disrupt splenic architecture and lead to the loss of marginal zone macrophages (MZMs) and marginal metalophillic macrophages (MMMs) (Beattie et al., 2006). As the spleen plays a vital role in the clearance of bacteria in the blood via these macrophage populations (Aichele et al., 2003; Lanoue et al., 2004; Aoshi et al., 2009), it led to the hypothesis that *P. yoelii* infection results in a loss of these cell populations and their absence contributes to impaired clearance of systemic bacterial infections. To test this hypothesis, mice were infected with Py and various days after infection spleens were removed for quantification of polymorphonuclear cells (PMNs), inflammatory monocytes, red pulp macrophages (RPMs), MZMs, and MMMs (Figure S1). As expected, the total cellularity of the spleen increased through resolution of detectable Py infected RBCs, followed by a return to baseline at 1- and 2-months post clearance of Py infected RBCs (Figure 3A). Analysis of PMNs revealed a consistent expansion in frequency (Figure 3B) and total number (Figure 3C) of PMNs at days 10, 30–33, and 65–67 post-infection. Whereas, the frequency of inflammatory monocytes remained consistent between Py-infected and control mice (Figure 3D), there was an increase in the total number of these cells at days 10 and 30–33 in Py-infected mice (Figure 3E). The frequency of RPMs was lower in Py-infected mice at days 30–33 and 65–67 post-infection (Figure 3F), yet the total cell numbers remained fairly consistent during acute parasitemia and following resolution of Py infected RBCs (Figure 3G). Consistent with the previous report (Beattie et al., 2006), we observed a decrease in the frequency (Figure 3H) and total number (Figure 3I) of MZMs at days 30–33, 65–67, and 101–102 post-Py infection; however, there was no difference at day 10 post-Py infection. In contrast, the frequency (Figure 3J) and total number (Figure 3K) of MMMs remained fairly consistent following Py infection. Collectively, there is no consistent increase or decrease in these cell populations that correlates with impaired clearance of Lm and Sp in Py-infected mice, suggesting that alternative mechanisms explain increased bacterial burden following Py infection.

### Contribution of *P. yoelii*-Induced HO-1 and IL-10 to Impaired Innate Immunity Is Bacteria-Dependent

Investigations of impaired immunity to non-Typhoid *Salmonella* (NTS) co-infections during malaria have reported that the activities of HO-1 and IL-10, which are produced during active *Plasmodium* infection, play an important role in the increased severity of bacterial infection (Cunnington et al., 2011; Lokken et al., 2014). To determine whether those mechanisms are specific to NTS co-infections, we first confirmed the results of those studies by infecting mice with *P. yoelii* followed by co-infection with NTS. Although not statistically significant, bacterial burdens in coinfected mice with tin protoporphyrin IX (Sn PP), whichh inhibits HO-I, trended lower than control. As shown before, *P. yoelii*-infected mice treated with an anti-IL-10 monoclonal antibody had reduced bacterial burdens compared to the respective control treated mice (Figure S2).

**Figure 4:**
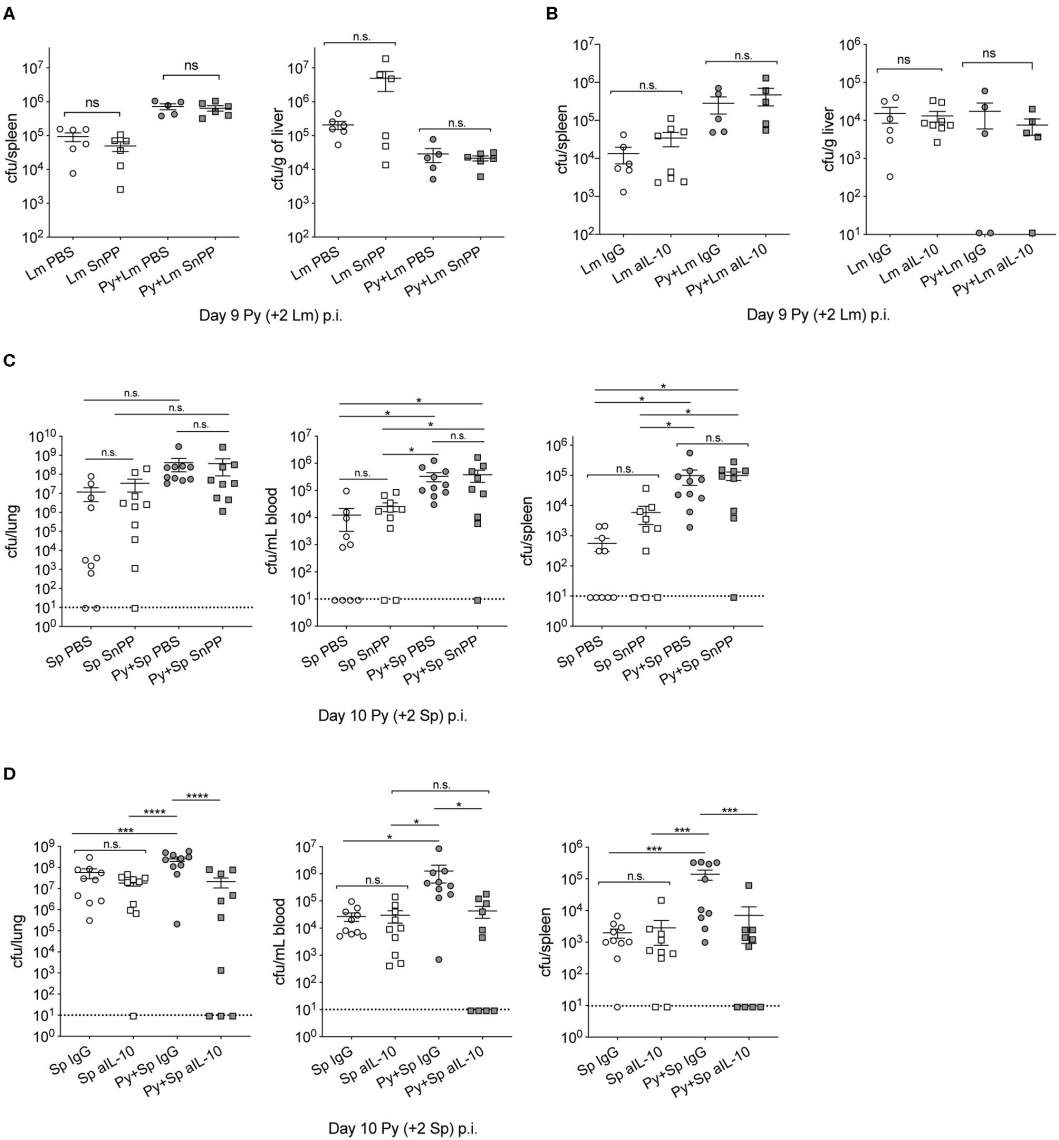
Effect of *P. yoelii* induced HO-1 and IL-10 on susceptibility to invasive bacterial infection is bacteria-specific. **(A,B)** C57BL/6N mice were infected with 10^5^ Py pRBCs, 7 days later both control and Py-infected mice were infected with 5 × 10^6^ CFU Lm. Spleen and liver bacterial burdens were determined day 2-post Lm infection. Mice were treated with tin protoporphyrin IX (Sn PP) or PBS control at 48, 24 and 8 h before Lm infection **(A)**, or αIL-10 antibody or isotype control on days 6, 7, and 8 post Py **(B)**. Data (mean ± S.E.) are cumulative results from two independent experiments with 3–6 mice per group analyzed with One-Way ANOVA followed by Tukey's multiple comparisons test. **(C,D)** C57BL/6N mice were infected with 10^5^ Py pRBCs, 8 days later both control and Py-infected mice were infected i.n. with 5 × 10^5^ CFU Sp. Lung, spleen, and peripheral blood bacterial burdens were determined day 2-post Sp infection. Mice were treated with tin protoporphyrin IX (Sn PP) or PBS control at 48, 24 and 8 h before Sp infection and 24 h post Sp infection **(C)**, or αIL-10 antibody or isotype control on days 6, 7, and 8 post Py **(D)**. Data (mean ± S.E.) are cumulative results from two independent experiments with five mice per group and analyzed with One-Way ANOVA followed by an uncorrected Fischer‘s LSD test with multiple comparisons, n.s., not significant. *p < 0.05, ***p < 0.001, and ****p < 0.0001.

Contrary to observations in NTS co-infections, Sn PP treatment did not improve anti-bacterial immunity in Py+Lm (Figure 4A) or Py+Sp (Figure 4C) co-infected groups, as bacterial burdens of Sn PP treated mice were comparable to control treated groups. Blocking IL-10 with an anti-IL-10 monoclonal antibody treatment prior to Lm infection did not improve bacterial clearance in co-infected mice (Figure 4B). In contrast, anti-IL-10 monoclonal antibody treatment in Py+Sp co-infected mice was sufficient to significantly reduce the severity of Pneumococcal infection compared to control IgG treated Py+Sp mice (Figure 4D). These data demonstrate that *Plasmodium*-induced IL-10 is part of the mechanism by which Py infection impairs control of Sp. The data also indicate that there may be additional mechanisms by which *Plasmodium* infections impair control of systemic bacterial infections.

### *P. yoelii* Impairs *Listeria*- and *Streptococcus*-Induced Reactive Oxygen Species in Neutrophils

**Figure 5:**
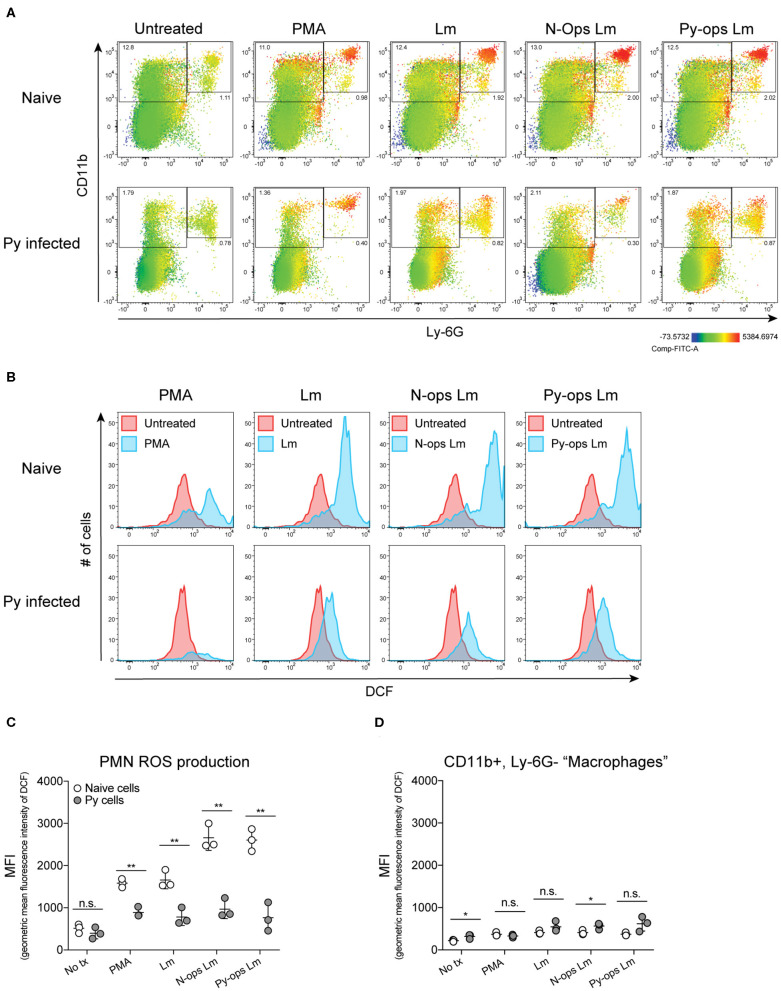
Splenic PMNs from *P. yoelii* infected mice have defective ROS production in response to Lm. C57BL/6N mice were infected with 10^5^ Py pRBCs. Single cell suspensions from whole spleens of naïve and Py-infected mice (days 14 or 15 p.i.) were incubated with DFC to detect intracellular ROS. Cells were then treated with PMA, Lm, or Lm opsonized with 10% serum from naïve or Py-infected mice. **(A)** Heat map displays DCF fluorescence in CD11b × Ly6G cells to indicate amount of intracellular ROS. Heat maps are from an individual mouse in each group that are representative of a single experiment and of three total independent experiments. **(B)** Histogram showing DCF fluorescence of splenic PMNs (CD11b^+^/Ly6G^+^) as shown in box in **(A)**. DCF geometric mean fluorescence intensity in PMNs (CD11b^+^/Ly6G^+^) **(C)** and macrophages/monocytes (CD11b^+^/Ly6G^−^) **(D)** populations as indicated in **(A)**. Each symbol represents an individual mouse. Data (mean ± S.D.) are representative of three total independent experiments with three mice per group. Statistical significance was determined with multiple unpaired *t*-tests. *p < 0.05 and **p < 0.01.

**Figure 6:**
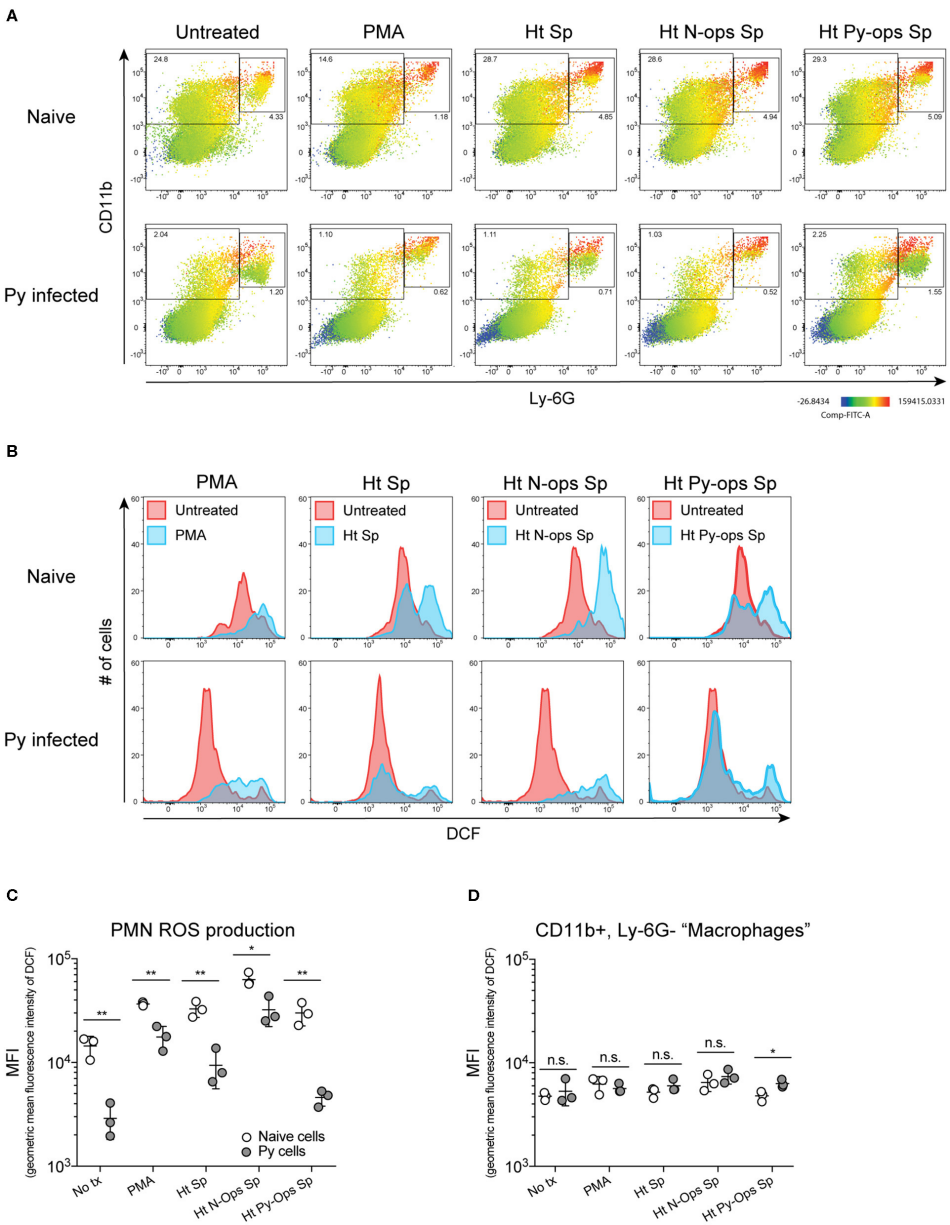
Splenic PMNs from *P. yoelii* infected mice have defective ROS production in response to heat-killed Sp. C57BL/6N mice were infected with 10^5^ Py pRBCs. Single cell suspensions from whole spleens of naïve and Py-infected mice (days 14 or 15 p.i.) were incubated with DFC to detect intracellular ROS. Cells were then treated with PMA, heat treated Sp (Ht Sp), or Ht Sp opsonized with 10% serum from naïve or Py-infected mice. **(A)** Heat map displays DCF fluorescence in CD11b × Ly6G cells to indicate amount of intracellular ROS. Heat maps are from an individual mouse in each group that are representative of a single experiment and of three total independent experiments. **(B)** Histogram showing DCF fluorescence of splenic PMNs (CD11b^+^/Ly6G^+^) as shown in box in **(A)**. DCF geometric mean fluorescence intensity in PMNs (CD11b^+^/Ly6G^+^) **(C)** and macrophages/monocytes (CD11b^+^/Ly6G^−^) **(D)** populations as indicated in **(A)**. Each symbol represents an individual mouse. Data (mean ± S.D.) are representative of three total independent experiments with three mice per group. Statistical significance was determined with multiple unpaired *t*-tests **(D)**. *p < 0.05 and **p < 0.01.

Production of reactive oxygen species (ROS) is an important antibacterial effector mechanism used by phagocytic cells that is necessary for immunity to a multitude of bacterial pathogens (Dinauer, 2005). To determine if impaired ROS production is a mechanism by which Py suppresses innate immunity during bacteremia, spleens were removed from naïve control and Py-infected mice 14–15 days post infection. ROS production was quantified in whole spleen single cell suspensions in untreated cells or cells stimulated with PMA, unopsonized Lm, Lm opsonized with serum from naïve mice (N-Ops Lm), or Lm opsonized with serum from Py-infected mice (Py-Ops Lm). In general ROS production was much more pronounced in Ly-6G^+^CD11b^+^ PMNs than Ly-6G^−^CD11b^+^ “macrophages” (Figure 5A). Consistent with increased Lm burden in Py-infected mice, PMNs from Py-infected mice showed decreases in intracellular ROS production (DCF geometric mean fluorescence intensity signal) in each of the stimulated groups (Figures 5B,C). In contrast, there was no difference in ROS production in Ly-6G^−^CD11b^+^ cells from Py-infected and naïve mice (Figure 5D). Similar results were also observed when splenocytes were stimulated with heat-treated (Ht) Sp, Ht N-ops Sp, or Ht Py-ops Sp (Figure 6).

Additional analysis of the data suggests that Py infection may impede immunity to Sp, at least in part, through a serum-dependent effect. As seen in Figure 6C, Ht Sp opsonized with serum from naive mice (Ht N-ops Sp) compared to Ht Sp opsonized with serum from Py-infected mice (Ht Py-ops Sp) elicited increased ROS production in PMNs from naïve mice (naïve cells treated with Ht N-Ops Sp vs. naïve cells treated with Ht Py-Ops Sp; open circle comparison in last two groups; unpaired *t*-test, *p* = 0.00087). An even more pronounced serum-dependent effect was observed in PMNs from Py-infected mice (Py cells Ht N-Ops Sp vs. Py cells Ht Py-Ops Sp; gray-filled circle comparison in last two groups; unpaired *t*-test, *p* = 0.00042). Of note, this serum-dependent effect was not observed following stimulation of Lm (Figure 5C). Collectively, these results identify impairment of ROS production in neutrophils as a potential mechanism by which Py suppresses antibacterial innate immunity during systemic bacterial infections, with an additional serum-dependent factor further hindering the cellular response to Sp.

### *Plasmodium*-Derived Hemozoin With Bound Biomolecules Suppresses Antibacterial Innate Immunity

**Figure 7:**
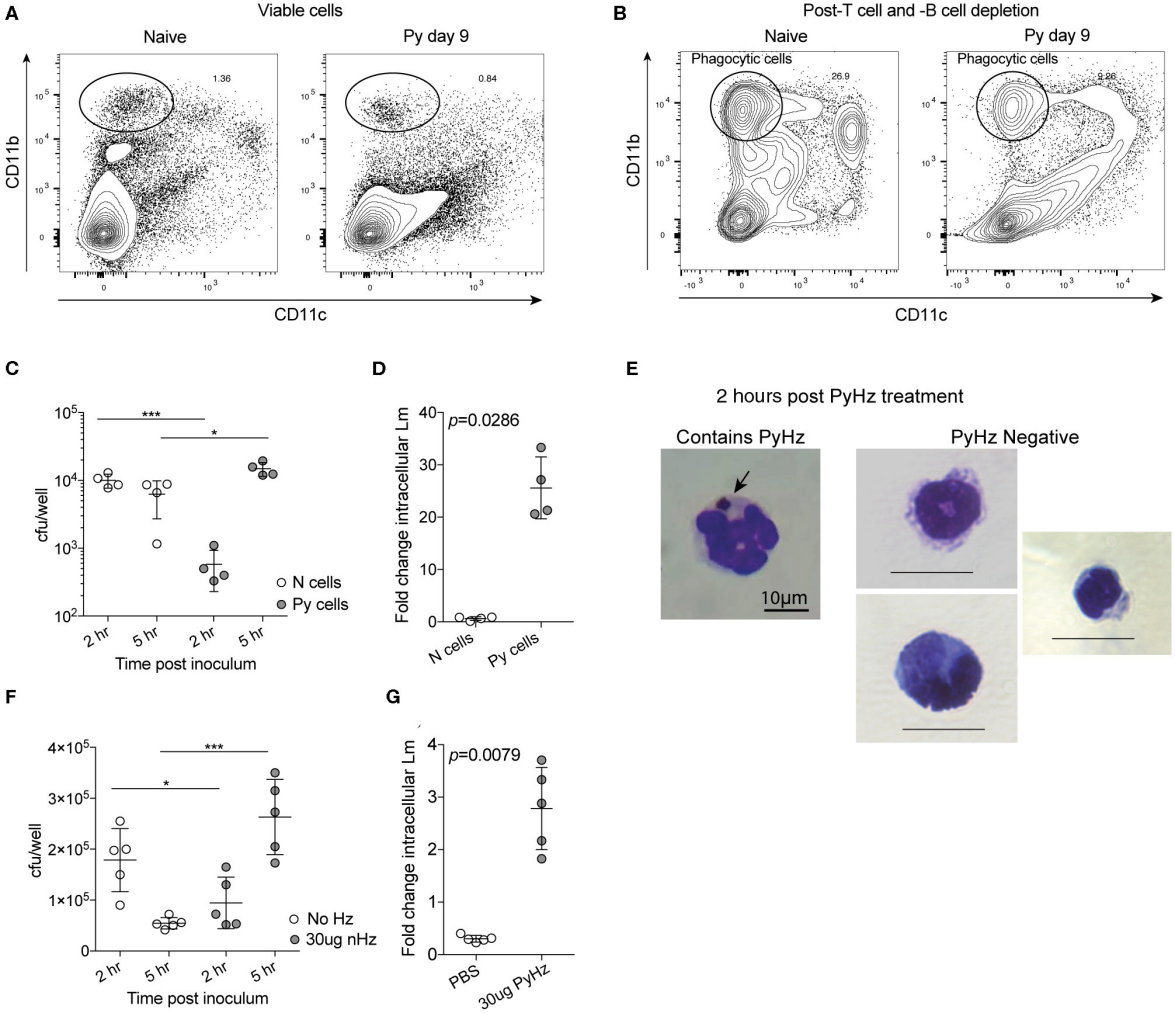
*P. yoelii* infection and parasite-derived hemozoin impairs anti-bacterial functions of splenic phagocytes. **(A–C)** After C57BL/6N mice were infected with 10^5^ Py pRBCs, splenic phagocytes were enriched from naïve or Py-infected mice day 9 post Py. Magnetic columns were used to enrich for splenic phagocytes by negative selection of CD3^+^CD19^+^ cells. **(A)** Contour plot showing CD11b and CD11c expression on viable splenocytes prior to T and B cell depletion on magnetic column. **(B)** Contour plot showing CD11b and CD11c expression on splenocytes following magnetic column depletion of T and B cells. Circle indicates the population of splenic phagocytes that was sort purified for gentamicin protection assays. CD11b^hi^CD11c^−^ cells were then sort purified for experiments. **(C,D)** 10^5^ sort-purified CD11b^hi^CD11c^−^ splenic phagocytes were plated in each well and infected with Lm opsonized with homologous mouse serum with MOI = 100. Intracellular Lm burden was determined after 2 and 5 h **(C)** in a gentamicin protection assay to evaluate fold change at 5 h post infection **(D)**. Fold change in intracellular bacteria was determined by cfu/well at 5 h post infection divided by cfu/well at 2 h post infection. **(E–G)** 10^5^ sort-purified CD11b^hi^CD11c^−^ splenic phagocytes were enriched, as in **(A,B)**, from naïve mice and were incubated with 30 μg hemozoin purified from spleen and livers of mice previously infected with Py (PyHz). **(E)** A giemsa-stained cell containing internalized PyHz (brown pigment), indicated by arrow, and PyHz negative cells containing no visible uptaken pigment. Intracellular Lm burden was determined after 2 and 5 h **(F)** in a gentamicin protection assay to evaluate fold change at 5 h post infection **(G)**. Fold change in intracellular bacteria was determined by cfu/well at 5 h post infection divided by cfu/well at 2 h post infection. Data (mean ± S.D.) are representative of three independent experiments each symbol represents an individual replicate of pooled cells from three mouse spleens per group (naïve or Py-infected). Statistical analysis was determined with Mann-Whitney test. *p < 0.05 and ***p < 0.001.

Decreased ROS production suggested that Py infection may create a permissive environment for invasive bacteria by suppressing antibacterial effector mechanisms of innate immune cells. To test the ability of innate immune cells to restrict intracellular bacterial growth, splenic phagocytes were first enriched from naïve control and Py-infected (day 9) mice via depletion of CD3^+^ and CD19^+^ cells (Figure 7A) and then CD11b^+^CD11c^−^ cells were sort purified (Figure 7B). Analysis of CD11b^+^CD11c^−^ splenocytes identified that about half were Ly-6G^+^ cells (Naïve = 43.9% ± 3.95; Py-infected day 10 = 52.28% ± 7.40), indicating mixed populations of PMNs and mononuclear cells in both groups. Sort purified CD11b^+^CD11c^−^ cells were infected with Lm in a 5-h gentamycin protection assay. Curiously, 2 h after infection cells from Py-infected mice had fewer bacteria than cells from naïve mice (Figure 7C). Whether this is due to differences in bacterial adherence, uptake, or killing at this time point is currently unknown. Importantly, 5 h after infection there was a significant increase in bacteria in cells from Py-infected mice compared to the 2-h time point as well as the naïve cell 5-h time point (Figure 7C), resulting in a significant difference in fold change in intracellular Lm between cells from naïve vs. Py-infected mice (Figure 7D). Similar results were also observed when phagocytes were sort-purified 29–35 days post-Py infection (data not shown).

Degradation of hemoglobin by *Plasmodium* results in heme, which is toxic to the parasite. To protect itself from heme, *Plasmodium* polymerizes heme into hemozoin (Hz), which is subsequently released into circulation when the parasite ruptures out of infected red blood cells (Francis et al., 1997; Pagola et al., 2000; Egan et al., 2002). Hz has been shown to remain in host tissues, including the spleen, for many months after the clearance of infection. This physical characteristic of Hz and the long-term suppression of innate immunity to bacteremia following clearance of Py infected RBCs (Figures 1C, 2F–K), led to the hypothesis that Hz is a parasite-derived factor that is capable of impairing anti-bacterial effector functions. To test this, Hz was extracted from spleens and livers of mice that had cleared Py infected RBCs (PyHz) and incubated with sort purified splenic phagocytes from naïve mice and then the cells were infected with Lm in a gentamycin protection assay. This Hz preparation preserved any associated molecules such as lipids and/or proteins, which are seen closely bound to Hz *in vivo* and have been implicated previously in its immunomodulatory properties (Schwarzer et al., 2003; Carney et al., 2006). Following incubation with PyHz, many CD11b^+^CD11c^−^ cells had phagocytosed PyHz, while others remained Hz negative (Figure 7E). Consistent with the hypothesis, PyHz impaired the ability of CD11b^+^CD11c^−^ cells to restrict the intracellular replication of Lm compared to PBS treated cells (Figures 7F,G). PyHz treated cells contained fewer Lm than untreated cells 2 h after infection, behaving similarly to splenocytes from Py-infected mice in this assay (Figure 7C). These data demonstrate that a parasite-derived factor (PyHz) was capable of impairing the antibacterial effector mechanisms of CD11b^+^CD11c^−^ cells in an *in vitro* assay.

**Figure 8:**
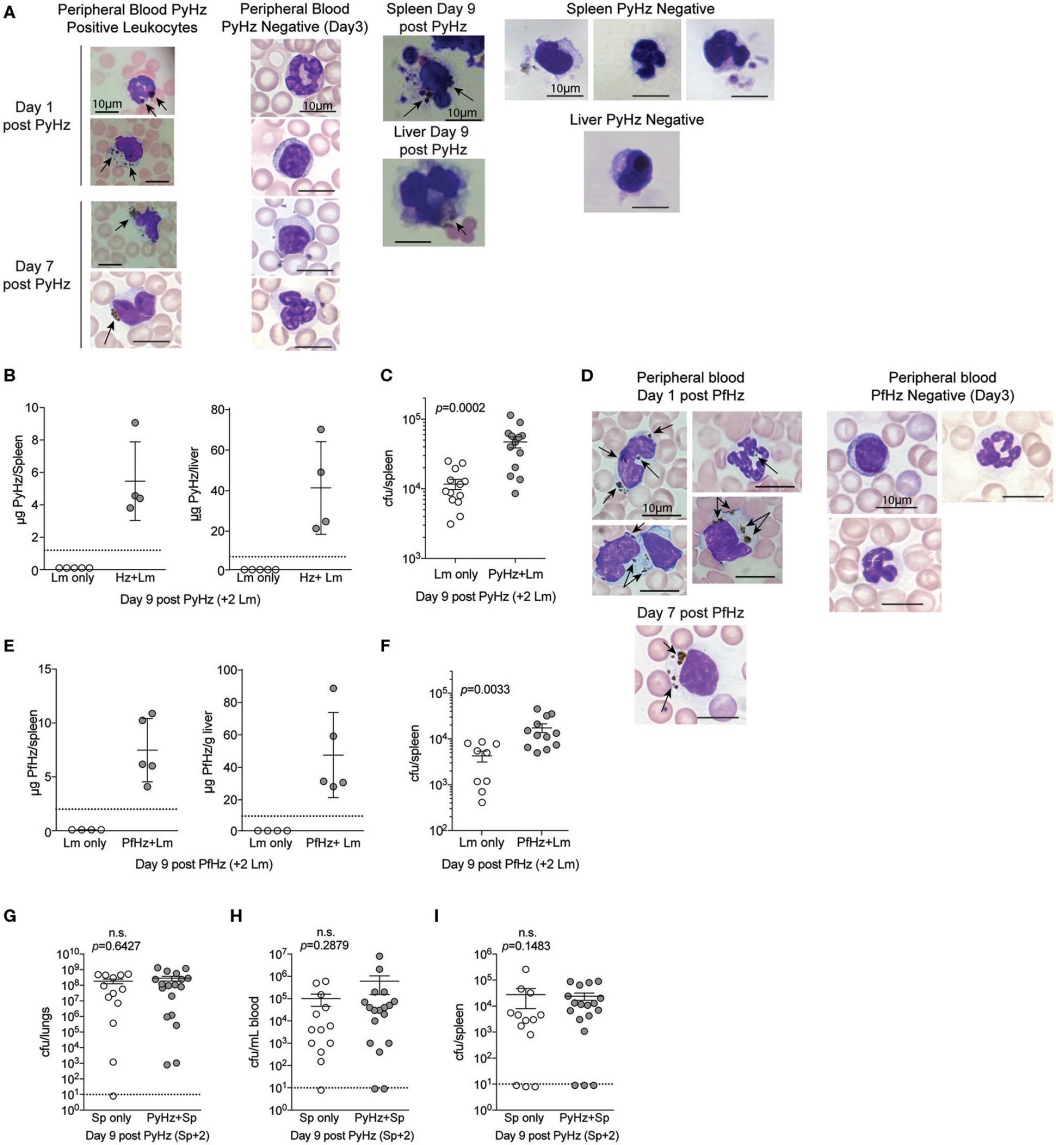
Hemozoin with bound biomolecules is sufficient to impair clearance of Lm *in vivo*. C57BL/6N mice treated i.v. with 1 mg PyHz isolated from livers and spleens of Py infected mice **(A–C)** or hemozoin isolated from *P. falciparum* (Pf) infected red blood cell culture (PfHz) **(D–F)**. Seven days after Hz treatment mice were infected with 5 × 10^6^ CFUs Lm. **(A,D)** Giemsa stained peripheral blood smears and cells mounted from spleen and liver single cell suspensions on the indicated days post Hz treatment. Arrows indicate the presence of intracellular Hz, visible as brown pigment. PyHz and PfHz-negative leukocytes were present at indicated time points in peripheral blood, spleen or liver with no visible intracellular Hz pigment **(B,E)**. Quantification of Hz in spleens and livers. Data (mean ± S.D.) are from a single experiment representing three independent experiments. Dotted lines represent the limit of Hz detection. Bacterial burden in spleens of PyHz **(C)**, PfHz **(F)** or untreated control mice was determined 2 days post Lm infection. Data (mean ± S.E.) are cumulative results from 2 to 3 independent experiments. Each symbol represents an individual mouse. **(G–I)** C57BL/6N mice were treated i.v. with 1–1.5mg PyHz followed by i.n. infection with 10^6^ CFU Sp 7 days later. Mice were sacrificed 2 days after Sp infection and lungs, blood, and spleens were collected for bacterial burden analysis. Data (mean ± S.E.) are pooled results from three independent experiments. For all data, each symbol represents an individual mouse. Statistical significance was determined with Mann-Whitney test.

To test whether PyHz was able to impair the clearance of Lm *in vivo*, naïve mice were given intravenous injections of PyHz followed by Lm infection 7-days later. PyHz was observed in circulating leukocytes both 1- and 7-days post injection as well as in spleen and liver cells 9-days after injection (Figure 8A). PyHz could also be quantified in both the spleen and liver 9-days post PyHz injection (Figure 8B). When Lm burden was quantified, PyHz treated mice had an elevated burden in the spleen (Figure 8C). Py is a rodent-specific species of *Plasmodium*, and previous studies exploring immune modulatory effects of Hz have typically isolated Hz from *Plasmodium falciparum* infected RBC cultures, rather than from organs of infected animal. To address potential differences in isolating Hz from organs of previously infected mice, and the ability of clinically relevant *P. falciparum* Hz to suppress innate immunity during bacteremia, Hz from *P. falciparum* infected red blood cell cultures (PfHz) was also tested. Consistent with Hz from Py infected mice, PfHz could be detected in the blood following injection (Figure 8D), was quantified in both the spleen and liver (Figure 8E), and PfHz treated mice had elevated bacterial burden 2-days post Lm infection (Figure 8F). Of note, PfHz was treated with DNase to remove DNA whereas PyHz had not been DNase treated. When PyHz was treated with DNase to remove DNA, mice injected with DNA free PyHz still exhibited elevated bacterial burden (Figures S3A–C). In contrast to these results, mice treated with “clean” PyHz, which was treated with detergents and proteinase K, followed by infection with Lm, showed no defect in the control of Lm (Figure S3D). Similarly, mice that were treated with pure synthetic Hz followed by Lm infection, also showed no defect in control of Lm (Figure S3E). Finally, PyHz treated mice infected with Sp, did not show different bacterial burdens in the lungs, peripheral blood, or spleen compared to control mice (Figures 8G–I). Collectively, these results identify Hz with bound bioactive molecules, excluding DNA, as a parasite-derived factor that is able to impair anti-bacterial innate immunity to some bacterial pathogens.

## Discussion

Identifying specific cell functions and broad, systemic deficiencies in innate immune responses during *Plasmodium* infections that are responsible for diminished bacterial immunity is necessary to improve the treatment of these cases. In this study, we sought to explore the underlying mechanisms of malaria-induced immune suppression and investigate what factors contribute to impaired innate immunity to invasive bacterial infection during both acute *Plasmodium* infection and following clearance of infected RBCs. Prior reports on NTS infections during malaria, demonstrated that infection-induced IL-10 production and infection-induced hemolysis, and activation of HO-1, were central to impaired immunity to NTS (Cunnington et al., 2011, 2012; Lokken et al., 2014). Here, we show that *Plasmodium*-induced IL-10 also enhances susceptibility to Sp co-infection and also demonstrated that *Plasmodium* infections can impair innate immunity to bacterial infections independent of HO-1 and IL-10. In our coinfection model with NTS we were not able to completely recapitulate results from Cunnington et al., where they found that inhibiting HO-1 with Sn PP reduced bacterial burdens in Py-infected mice. While NTS burdens trended lower in coinfected mice treated with Sn PP in our model system, differences between treated and untreated control groups were not statistically significant (Figure S2A). This may be due to technical differences in our bacterial inoculum or preparation. Thus, more work is needed to evaluate the possible role of HO-1 in our Lm and Sp malaria coinfection models.

It has been postulated that *Plasmodium*-induced immune suppression and susceptibility to invasive bacterial infections is not attributed to a parasite-specific factor. Rather, it is due to *Plasmodium*-induced hemolysis, and subsequent activation of HO-1 and IL-10 that inhibit neutrophil functions lead to weakened immune responses to invasive bacterial infections (Mooney et al., 2019). Yet, we demonstrate that Py-induced immune suppression lasts up to 2-months post clearance of Py infected RBCs, when the effects of hemolysis are likely no longer in play. Additionally, Cunnington and colleagues demonstrated that neutrophils collected from patients previously infected with *P. falciparum* had defective ROS burst following PMA stimulation that lasted up to 8 weeks-post treatment (Cunnington et al., 2012). Of note, Hz from *P. berghei* infection remains deposited in liver, bone marrow, and spleen tissues as well as within isolated CD11b^+^ cells isolated from those tissues and peripheral blood of mice for at least 196 days post infection (Frita et al., 2012). Collectively, these data provide evidence that a parasite factor, Hz, may contribute to this impaired innate immunity to bacterial co-infections and leads to the hypothesis that this factor contributes to clinical observations of reduced immunity to invasive bacterial infections in patients with history of malaria.

Using a murine infection model of malaria and *ex vivo* intracellular bacterial survival assays we have determined that Hz is able to impair innate immunity to bacterial infections independent of the parasite infection. Of note, we made this observation using two different preparations of Hz. First, Hz was prepared via a novel method by extracting Hz from organs of mice previously infected with Py. Importantly, DNase treated PyHz, to eliminate parasite and mouse DNA, was still capable of suppressing innate immunity to Lm *in vivo* (Figures S3A–C). Second, we demonstrated natural PfHz prepared from infected red blood cell cultures (Barrera et al., 2011) was able to suppress innate immunity *in vivo*. These data demonstrate that the immune modulatory effect of PyHz is unlikely due to parasite or mouse DNA. Whereas, parasite or mouse DNA were not implicated in the immune suppressive nature of PyHz, PyHz prepared in the presence of detergents and proteases (Figure S3D), as well as synthetic Hz (Figure S3E) did not impair *in vivo* innate immune responses. This supports the idea demonstrated by Carney et al. that the immune modulatory effect of Hz is perhaps not due to Hz itself or adherent DNA, but attributed to interactions with RBC membrane lipids and other debris that subsequently generate highly reactive secondary metabolic products capable of disrupting cell functions (e.g., NADPH oxidase and iNOS) upon uptake (Carney et al., 2006). Furthermore, lipids within parasitized RBCs and hemozoin, such as the hydroxy fatty acids, hydroxy-arachidonic acid (HETE) and hydroxy-linoleic acid (HODE), have been shown to inhibit PMA-stimulated reactive oxygen burst in human peripheral blood monocytes (Schwarzer et al., 2003).

Our results suggest the possibility that parasite-derived Hz and bound molecules are contributing to the impaired innate immune response to Lm, potentially through inhibition of ROS production. While our data indicate that Hz impairs immunity to Lm, our “native” preparations of PyHz did not impair immunity to Sp in our model system. There are two potential explanations for this observation. First, intravenous injection of PyHz may not have resulted in sufficient levels of Hz to suppress innate immunity to Sp. This possibility is supported by the observation that Py infection impairs innate immunity to Sp long after the clearance of Py infected RBCs (Figures 2I–K), suggesting a bona fide role for Hz in this immune suppression. Second, differences in Hz-driven impaired innate immunity between Lm and Sp may be explained by the intracellular and extracellular nature of these pathogens, respectively. Lm is considered an “intracellular pathogen” since it grows within cells, whereas Sp is generally thought of as extracellular. Although this is not exclusive as Sp can grow in CD169^+^ splenic macrophages (Ercoli et al., 2018).

Our *in vitro* data demonstrates the impaired anti-bacterial ability of splenic phagocytes either isolated from Py-infected mice or treated with PyHz. Interestingly, the analysis of intracellular cfu burdens in our *in vitro* assay found that splenic phagocytes from Py-infected mice, or phagocytes treated with PyHz, contained significantly less Lm at 2 h post infection (with 1 h in gentamicin) compared to cells from naïve mice or untreated cells (Figures 7C,F). There are several explanations for this result, including a defect in Lm uptake, differences in Lm adherence, and altered killing of intracellular or extracellular Lm during the time prior to the 2-h time point. Additional work will be needed to further understand the impact of Py-infection and hemozoin in these interactions. Furthermore, cfu counts at 5 h post infection suggest that PyHz treated or Py-infected mouse phagocytes exhibit a profound inability to restrict intracellular bacterial growth. In contrast, there may be a different mechanism responsible for *Plasmodium*-dependent susceptibility to extracellular bacteria. For example, serum-dependent effects as we observed (Figure 6C). This is discussed below in more detail. Further studies are required to elucidate the mechanisms underlying susceptibility to Sp and other extracellular bacterial pathogens during co-infection with *Plasmodium*. Methods that can recognize the presence or absence of internalized Hz at the single cell level would substantially enhance our understanding of this interaction. Additional work is also needed to uncover the immune cell types affected as well as the antimicrobial functions that are impaired by *Plasmodium* in the context of Sp and other clinically important co-infections during malaria.

Hz may directly impair antibacterial effector mechanisms of innate immune cells via uptake or interaction with Hz. The uptake and recognition of Hz by phagocytic cells could have an indirect effect through altering the host microenvironment via the induction of pathways that influence the production of cytokines and chemokines in a manner that creates a more favorable environment for invasive/pathogenic bacteria. Hz has been shown to induce trained immunity (Schrum et al., 2018), suggesting the possibility that long-term suppression of innate immunity to systemic bacterial infections may in part be attributed Hz-induced epigenetic modifications. CD317^+^ splenic dendritic cells purified day 7 post-Py or -*Plasmodium chabaudi* infection have been shown to be sufficient at transferring infection to naïve mice (Wykes et al., 2011). Additionally, CD317^+^ dendritic cells purified after initial clearance of patent *P. chabaudi* infection, just before recrudescent parasitemia (about 3-weeks post-infection), were also sufficient at causing infection in naïve mice. While the presence of replicative parasites surviving within splenic DCs has not been described after resolution of acute hyperparasitemia in Py infection, it is possible that persistent, undetectable parasites in tissues or circulation may be contributing to the long-term impaired immunity we describe in our Py bacterial coinfection models.

Clinical reports of invasive bacterial disease coinciding with malaria often report both NTS and Sp as the two organisms most commonly isolated from blood. Experimental studies to date have focused almost exclusively on NTS and it is unknown whether the mechanisms of *Plasmodium*-induced immune suppression to NTS will be important for Sp or other bacterial pathogens found in co-infections with malaria (e.g., *Haemophilus influenzae, Mycobacterium tuberculosis, Staphylococcus aureus, Streptococcus pyogenes*, etc. Berkley et al., 2005; Reddy et al., 2010; Colombatti et al., 2011; Scott et al., 2011; Were et al., 2011; Auma et al., 2013; Gomez-Perez et al., 2014; Mourembou et al., 2016; Morton et al., 2018). We report for the first time, to our knowledge, a murine *Plasmodium* and pneumococcal co-infection. Mice were infected with *Plasmodium yoelii* 17XNL followed by a *Streptococcus pneumoniae* ATCC #6303, a type 3 capsule serotype strain, via the natural intranasal route. Consistent with the risk of invasive Sp infections in humans with malaria (Scott et al., 2011), we found decreased survival and increased bacterial burdens in co-infected mice compared to control Sp only infected mice. Murine models of malaria associated-respiratory distress syndrome (MA-ARDS) using *P. berghei* NK65 strain have found an association between pulmonary pathology and the levels of Hz deposition (Deroost et al., 2013). Pulmonary pathology matching MA-ARDS has been reported in Py 17X but not Py 17XNL infection (Fu et al., 2012); however, the parasitemia obtained in mice infected with Py 17XNL in that study were much lower than what was observed in this report where Py 17XNL parasitemia reached 40–60%. Consequently, Hz deposition in the lung, and potentially in spleen and other organs, may contribute to differential invasiveness of Sp during *Plasmodium* infection. Further studies are necessary to determine to what degree *Plasmodium*, via Hz or other mechanisms, induces lung pathology and to what degree this contributes to increased susceptibility to invasive Sp infection.

In addition to impaired ROS production, Py infection may also impair the innate immune response to invasive bacteria via serum-dependent effects. Complement components C1q and C3 interact with parasitized RBCs during *P. falciparum* infection, initiating the complement cascade, which leads to reduced serum levels of C1q and lower complement activity compared to uninfected individuals (Nyakoe et al., 2009; Silver et al., 2010). Several components of the complement system are considered essential for immunity to Sp infection, as individuals with genetic defects in C3, which is involved in both the classical and alternative complement pathways, experience recurring pneumococcal infections and infections with other encapsulated bacteria. Similarly, mice lacking expression of *C3*, factor B, *C1qa, or C4* genes are more susceptible to Sp infections (Brown et al., 2002). Moreover, killing of Sp by neutrophils has been shown to require active complement opsonophagocytosis via CR3 (CD11b/CD18 heterodimer complement receptor; Standish and Weiser, 2009). Consistent with *Plasmodium*-induced depletion of complement and a role for complement in immunity to Sp, we observed decreased ROS production in splenic PMNs from naïve mice stimulated with heat-killed Sp opsonized with serum from Py-infected mice compared to splenic PMNs stimulated with heat-killed Sp opsonized with serum from naïve mice (Figures 6A–C). Similarly, PMNs from Py-infected mice showed increased ROS production following stimulation with heat-killed Sp opsonized with serum from naïve mice compared to serum from Py-infected mice. This observation could indicate a serum factor-dependent defect in neutrophil opsonophagocytosis that may lead to impaired immunity to Sp during malaria. Although ROS bursts within neutrophil phagolysosomes are not sufficient to kill Sp (Standish and Weiser, 2009), deficiency in PMN ROS production in response to heat-killed Sp opsonized with serum from Py-infected mice compared to heat-killed Sp opsonized with serum from naïve mice suggests that phagolysosome maturation, or other events that follow opsonophagocytosis, may be inhibited or less responsive. It has also been shown that serum from febrile *P. falciparum* infected individuals results in decreased C3 deposition on NTS compared to serum from non-febrile *P. falciparum* negative individuals (Nyirenda et al., 2018). Collectively, these observations suggest that complement depletion during *Plasmodium* infections may be a general deficiency associated with invasive bacterial infections.

The impact of hemolysis-related immune suppression initiated by *Plasmodium* infection (activation of HO-1 and IL-10) has been well studied in the context of NTS in both animal models and clinical studies (Cunnington et al., 2012; Lokken et al., 2014; Mooney et al., 2018). By exploring co-infection models with different bacterial pathogens during malaria, we sought to determine whether the IL-10-dependent and HO-1-dependent mechanisms were specific to NTS co-infection, or if they also play a role in malaria-related immunosuppression to other bacterial co-infections. While our results indicated that HO-1 inhibition with Sn PP failed to improve immunity to either Lm or Sp co-infections (Figures 4A,C), we did see a reduction in Sp bacterial burdens in co-infected mice treated with anti-IL-10 (blocking) antibody (Figure 4D). This result is in line with observations made in influenza and pneumococcal co-infections, where IL-10 production following influenza virus infection disrupts the inflammatory response to Sp in a manner that favors dissemination and invasive disease (van der Sluijs et al., 2004). Blocking IL-10 activity in the Py+Sp co-infection may, similarly to what is seen in the influenza co-infection (van der Sluijs et al., 2004), result in increased levels of inflammatory factors in the respiratory tract, such as TNFα and IFNγ, enhanced PMN infiltration to lungs, and restore the antibacterial immune response to Sp to normal. IL-10 is known to impair neutrophil chemotaxis, which may contribute to “neutrophil paralysis” that has been characterized in samples from patients with *P. vivax* malaria (Leoratti et al., 2012). Thus, the anti-IL-10 treatments may have restored neutrophil function, resulting the reduction in pneumococcal burden in anti-IL-10 treated co-infected mice compared to isotype-treated controls. While this result shows that IL-10 is involved in suppressing the immune response to Sp during Py infection, it likely does not explain the long-term impaired immunity to Sp we have demonstrated (Figures 2F–K), since levels of IL-10 decrease following parasite clearance (Peyron et al., 1994).

This work provides evidence that *Plasmodium* infections induce long-term, hemolysis-independent, impaired immunity to bacterial co-infections and that a parasite factor, hemozoin with bound bioactive molecules, impairs *in vivo* clearance of systemic bacterial infections. Additionally, we find that multiple *Plasmodium-*induced mechanisms contribute to susceptibility to bacterial co-infections, with specific mechanisms dependent on the particular bacterial pathogen. It is also possible that the mechanisms of *Plasmodium-*impaired immune functions impact different stages in various bacterial infections. Differences in the three bacterial infections modeled in this work, intravenous, intranasal and intraperitoneal inoculations for Lm, Sp, and NTS, respectively, relate to drastically different progressions of infection, featuring different levels of immune system bottlenecks. The stage at which each bacterial pathogen is able to take advantage of these Py-dependent impaired immune functions to achieve an enhanced infection are therefore likely to differ depending on the pathogen.

There are currently no effective malaria vaccines that provide long lasting protection, and the range of malaria transmission by female *Anopheles* mosquitoes is predicted to increase with respect to global climate change (Hertig, 2019). Therefore, the contribution of hemozoin and impaired serum components to bacterial infection need be reevaluated and investigated experimentally to improve our understanding and treatment of invasive bacterial infection associated with malaria. In particular, the duration of Hz-dependent impaired innate immunity following *Plasmodium* infection needs to be assessed so that a history of malaria, rather than a positive blood smear during an active infection, can be considered when assessing a patient's risk of developing an invasive bacterial infection to avoid post-discharge morbidity.

## Data Availability Statement

The raw data supporting the conclusions of this article will be made available by the authors, without undue reservation.

## Ethics Statement

The animal study was reviewed and approved by the University of Louisville Institutional Animal Care and Use Committee and Indiana University Institutional Animal Care and Use Committee.

## Author Contributions

CH and NS designed and performed experiments, analyzed data, drafted, reviewed, and edited the manuscript. NV performed experiments, analyzed data, reviewed, and edited the manuscript. EV prepared PfHz and reviewed the manuscript. ES provided PfHz, reviewed, and edited the manuscript. All authors contributed to the article and approved the submitted version.

## Conflict of Interest

The authors declare that the research was conducted in the absence of any commercial or financial relationships that could be construed as a potential conflict of interest.
