# Supplementary material for: Plasmodium Impairs Antibacterial Innate Immunity to Systemic Infections in Part Through Hemozoin-Bound Bioactive Molecules
Source: Front Cell Infect Microbiol. 2020 Jun 30;10:328. doi: 10.3389/fcimb.2020.00328 (PMC7344233; doi:10.3389/fcimb.2020.00328)
Supplement: Supplementary file 1 [file Data_Sheet_1.PDF]

Supplementary Materials

Plasmodium impairs antibacterial innate immunity to systemic infections in part through hemozoin-bound bioactive molecules

Christopher L. Harding[1], Nicolas F. Villarino[2], Elena Valente[3], Evelin Schwarzer[3], and Nathan W. Schmidt[1,4‡*]

[1]Department of Microbiology and Immunology, University of Louisville, Louisville, KY, USA.
[2]Department of Veterinary Clinical Sciences, Washington State University, Pullman, WA, USA.
[3]Department of Oncology, University of Torino, Torino, Italy.
[4]Ryan White Center for Pediatric Infectious Diseases and Global Health, Department of Pediatrics, Indiana University School of Medicine, Indianapolis, IN, USA.

*** Correspondence:**
Nathan W. Schmidt
nwschmid@iu.edu
[‡]Current address: Ryan White Center for Pediatric Infectious Diseases and Global Health, Department of Pediatrics, Indiana University School of Medicine, Indianapolis, IN, USA.

Supplementary Methods

Infections

Salmonella enterica serovar Typhimurium (NTS): Frozen aliquots of *Salmonella enterica* serovar Typhimurium strain 12023 were thawed and resuspended in LB broth and grown shaking at 37˚C. Cultures were then washed in sterile 1X PBS and resuspended in sterile saline. Mice were infected on day 10 post Py infection via intraperitoneal injection of $1x10^3$ cfu in 200µl. Inoculum dose was verified by plating serial dilutions on LB agar containing 50µg/mL carbenicillin. For analysis of bacterial burdens in NTS infections, peripheral blood and organ homogenates were plated in serial dilutions on LB agar plates containing 50µg/mL carbenicillin.

In vivo Inhibition of IL-10 and HO-1

Py-infected and naïve mice were injected i.p. with 300, 200, and 100µg of rat anti-IL-10 IgG1 kapa monoclonal antibody (clone JES5-2A5) or rat anti-horseradish peroxidase IgG1 isotype control (In*Vivo*MAb, West Lebanon, NH) in 0.2 mL sterile saline on days Py+9, 10, and 11 (days NTS -1, 0, +1) for NTS co-infections. Similarly, Py-infected and naïve mice were treated i.p. with 40µmol/kg Tin Protoporphyrin IX (chloride) (Sn PP) (Cayman Chemical, Ann Arbor, MI) in 0.2mL 1X PBS on days Py+8, +9, 10, and 11 (days NTS -2, -1, 0, +1) for NTS co-infections.

Hemozoin Treatments

Mice were injected with 1mg of DNase treated PyHz, PyHz treated with detergent, DNase, and Proteinase K ('clean'Hz), or synthetic hemozoin (sHz) (InVivoGen, San Diego, CA) various preparations of hemozoin (described in materials and methods section) intravenously. Clean PyHz was prepared for some experiments with a method adapted from Coban et al. (Coban et al., 2002), where organ homogenate was lysed in 0.2% IGPAL and washed 3-4 times in 2% sodium dodecyl sulfate (SDS) and then incubated overnight in 10mM Tris-HCl (pH 8.0), 0.5% SDS, 1mM $CaCl_2$, and 2mg/mL proteinase K at 37˚C. Next, samples were washed again in 2% SDS and then incubated at 37˚C for 3 hours in 6M urea, washed in 1X PBS and resuspended in saline for experimental use. Immediately before Hz injections, all preparations were passed through a series of syringes with needles of increasing gauge, from 18G to 32G, in order to break up large pieces and avoid harming the mice. Between 9- and 20-days post Hz treatment, mice were infected with $5x10^6$ CFU *Listeria monocytogenes* (Lm). Organs from Hz treated, and untreated control mice were harvested 2- or 3-days post Lm infection.

Supplementary Figures

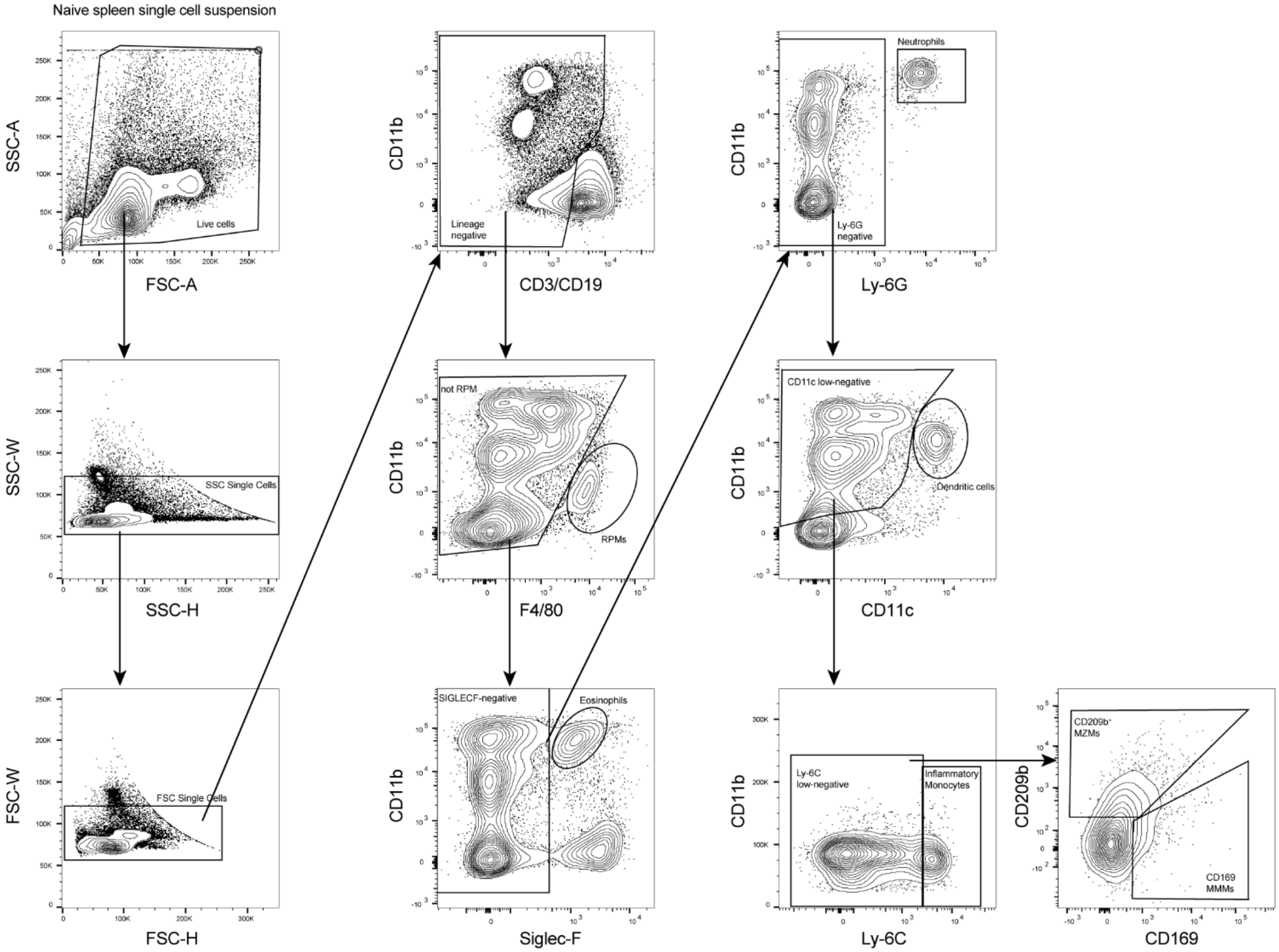

Supplementary Figure 1. Gating strategy for splenic myeloid cell populations. Single cell suspensions of spleens collected from mice infected with Py or age-matched naïve controls were prepared and stained with antibodies for flow cytometry analysis. Representative contour plots are from a naïve mouse. Arrows represent the hierarchical gating strategy that was used to determine the indicated cell populations. RPMs were gated early to avoid misidentification due to the highly auto-fluorescent nature of this cell type (Franken et al., 2015).

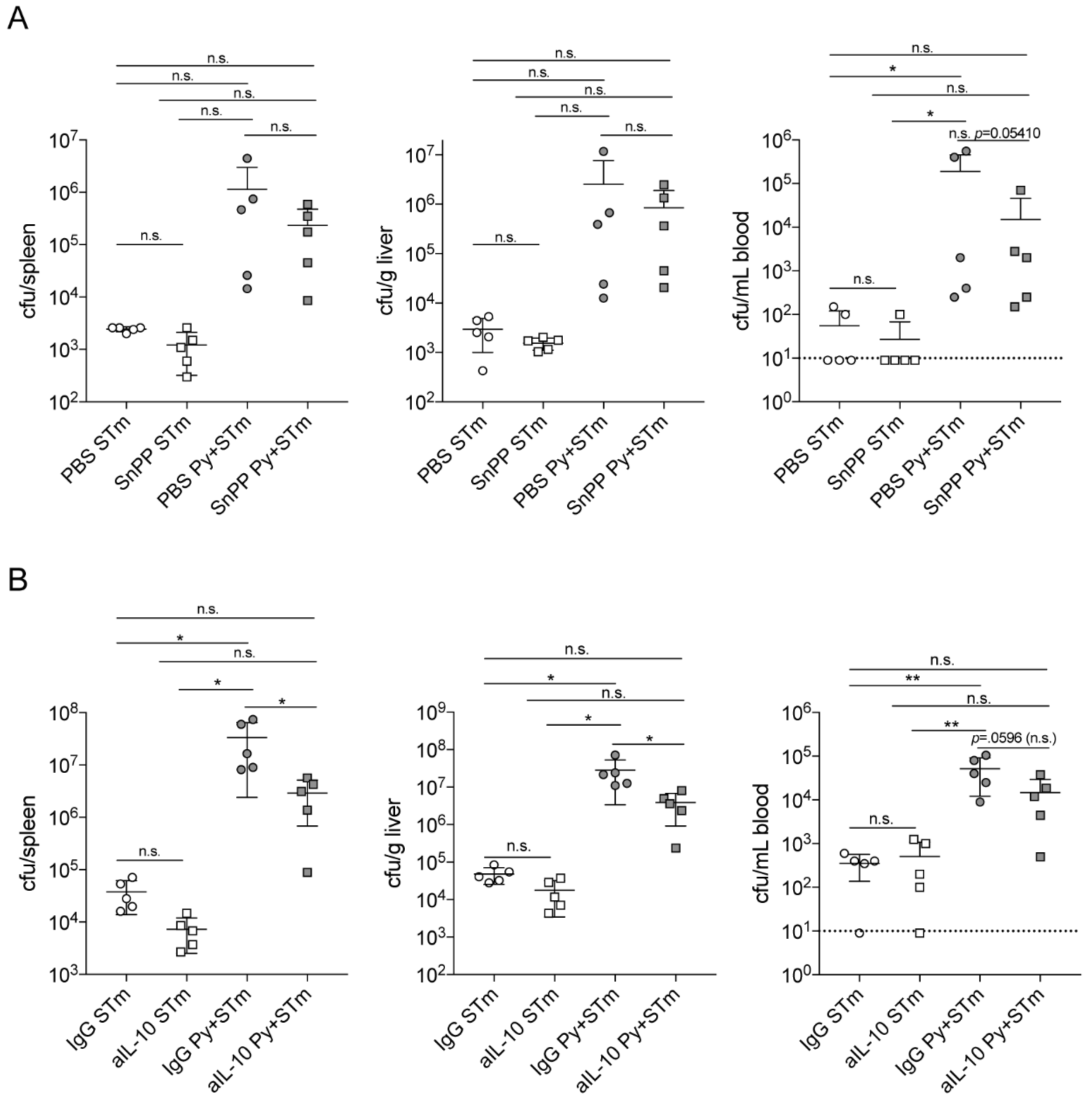

Supplemental Figure 2. Inhibition of HO-1 and IL-10 decreases bacterial burdens in *P. yoelii* non-Typhoid *Salmonella* co-infected mice.

C57BL/6N mice were infected with 10^5 Py pRBCs, ten days later both control and Py-infected mice were infected via intraperitoneal injection with 10^3 CFU *Salmonella enterica* serovar Typhimurium (NTS). Spleen, liver, and peripheral blood bacterial burdens were determined day 2-post NTS infection. Mice were treated with tin protoporphyrin IX (Sn PP) or PBS control at 48, 24 and 8 hours before NTS infection and 18 hours post-NTS infection (A), or anti-IL-10 antibody or IgG isotype control on days 9, 10, and 11 post Py (B). Data (mean±S.D.) are representative of two independent experiments with 5 mice per group (A) or are from a single experiment with 5 mice per group (B).

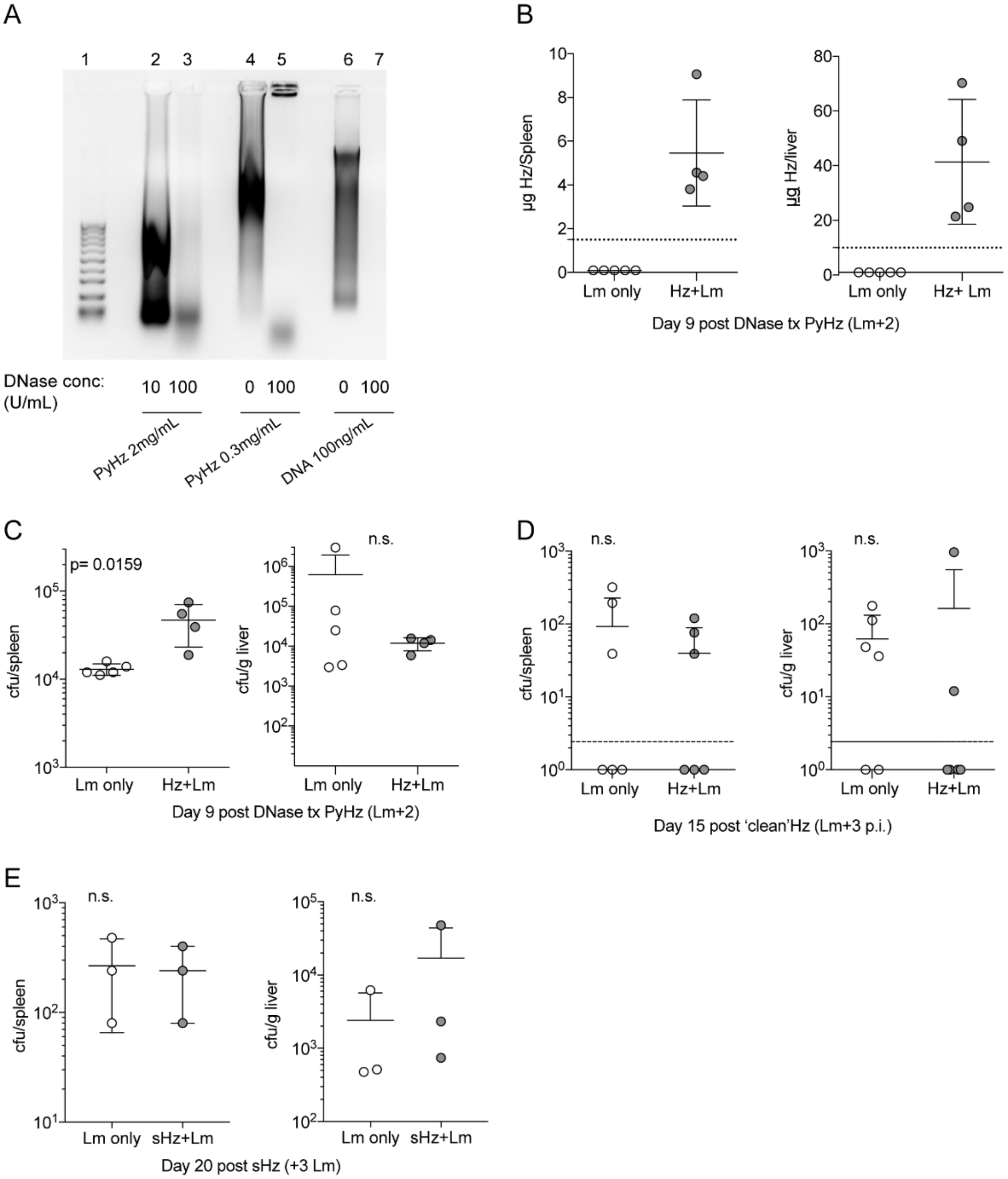

Supplemental Figure 3. Hemozoin preperation differentially impacts anti-bacterial innate immunity. A) Agarose gel containing PyHz samples labeled with GelRed Nucleic Acid stain to detect presence of DNA. Lane 1 – 1,000-100 base pair ladder, lane 2 – 15µL sample of 2mg/mL PyHz treated with 10 U/mL DNase, lane 3 – 15µL sample of 2mg/mL PyHz treated with 100 U/mL DNase, lane 4 – 15 µL sample of untreated 0.3 mg/mL PyHz, lane 5 – 15 µL sample of 0.3 mg/mL PyHz treated with 100U/mL DNase, lane 6 – 15µL of 100ng/mL untreated DNA, lane 7 – 15µL of 100ng/mL DNA treated with 100U/mL DNase. B) Quantification of DNase treated PyHz in spleens and livers. Data (mean±S.D.) are representative of three independent experiments. Dotted lines represent the limit of Hz detection. C-E) Lm burdens in spleens and livers from mice treated with DNase treated Hz (C), 'clean' PyHz (D), and synthetic Hz (E) on the indicated days post Hz treatment and Lm infection. Data were analyzed by Mann Whitney test.

References

Coban, C., Ishii, K.J., Sullivan, D.J., and Kumar, N. (2002). Purified malaria pigment (hemozoin) enhances dendritic cell maturation and modulates the isotype of antibodies induced by a DNA vaccine. *Infect Immun* 70(7)**,** 3939-3943. doi: 10.1128/iai.70.7.3939-3943.2002.

Franken, L., Klein, M., Spasova, M., Elsukova, A., Wiedwald, U., Welz, M., et al. (2015). Splenic red pulp macrophages are intrinsically superparamagnetic and contaminate magnetic cell isolates. *Sci Rep* 5**,** 12940. doi: 10.1038/srep12940.
